# A 3D self-organizing multicellular epidermis model of barrier formation and hydration with realistic cell morphology based on EPISIM

**DOI:** 10.1038/srep43472

**Published:** 2017-03-06

**Authors:** Thomas Sütterlin, Erika Tsingos, Jalil Bensaci, Georgios N. Stamatas, Niels Grabe

**Affiliations:** 1 Hamamatsu TIGA Center, BioQuant, Heidelberg University, Im Neuenheimer Feld 267, 69120 Heidelberg, Germany; 2 National Center for Tumor Diseases, Dept. of Medical Oncology, Im Neuenheimer Feld 460, 69120 Heidelberg, Germany; 3 Centre for Organismal Studies, Heidelberg University, Im Neuenheimer Feld 230, 69120 Heidelberg, Germany; 4 Emerging Science & Innovation, Johnson & Johnson, Santé Beauté France, 1 rue Camille Desmoulins, 92130 Issy les Moulineaux, France

## Abstract

The epidermis and the stratum corneum (SC) as its outermost layer have evolved to protect the body from evaporative water loss to the environment. To morphologically represent the extremely flattened cells of the SC - and thereby the epidermal barrier - in a multicellular computational model, we developed a 3D biomechanical model (BM) based on ellipsoid cell shapes. We integrated the BM in the multicellular modelling and simulation platform EPISIM. We created a cell behavioural model (CBM) with EPISIM encompassing regulatory feedback loops between the epidermal barrier, water loss to the environment, and water and calcium flow within the tissue. This CBM allows a small number of stem cells to initiate self-organizing epidermal stratification, yielding the spontaneous emergence of water and calcium gradients comparable to experimental data. We find that the 3D *in silico* epidermis attains homeostasis most quickly at high ambient humidity, and once in homeostasis the epidermal barrier robustly buffers changes in humidity. Our model yields an *in silico* epidermis with a previously unattained realistic morphology, whose cell neighbour topology is validated with experimental data obtained from *in vivo* images. This work paves the way to computationally investigate how an impaired SC barrier precipitates disease.

Protection against dehydration is the foremost function of the epidermis, a complex 3D tissue containing multiple cells types that differ in function and morphology^1^,^2^. The outermost epidermal cells form a protective layer, the stratum corneum (SC), which creates a barrier to the diffusion of water. In the absence of this epidermal barrier, water rapidly evaporates with fatal consequences^3^,^4^. The stratum granulosum (SG), also contributes to the epidermal barrier^5^,^6^. Corneocytes, the cells in the SC, have a distinctive flat shape that increases tissue tortuosity, thus reducing diffusion and transepidermal water loss (TEWL)^7^,^8^. Smaller and rounder corneocytes are found in the sensitive skin of infants^9^,^10^, and in individuals affected by atopic dermatitis or psoriasis^11^. Skin hydration regulates the differentiation of corneocytes, thus impacting physical skin appearance, epidermal homeostasis, and the barrier function^1^. Similarly, epidermal calcium content affects cell differentiation in the epidermis^12^,^13^. Therefore, the interplay of the epidermal barrier with epidermal water and calcium content plays an important role in skin care.

Systems biology offers a framework to comprehend complex biological systems, such as multifactorial diseases that have thus far eluded molecular biology approaches^14^. Skin research has much to gain from advances in computational modelling, particularly as practical and ethical barriers limit invasive experiments. Ideally, a computational model of epidermis would allow physicians to monitor a patient-specific 3D dynamic epidermis *in silico* without compromising its integrity, allowing insights impossible to obtain *in vivo* without invasive procedures. In this work, we create a proof-of-principle computational model of human epidermal homeostasis with realistic morphology that incorporates the epidermal barrier and epidermal calcium and water gradients. In particular, we aimed to develop a model that reproduced these features as an emergent property using existing knowledge from literature.

Healthy epidermal homeostasis entails constant turnover of individual cells that change their shape and function as they move through the epidermal layers^1^. We use a cell-centred agent-based modelling approach to encompass the life-history of individual cells in their 3D environment. This approach has been used previously to model several aspects of epidermal biology, ranging from simple keratinocyte cultures^15^,^16^, to models of tissue homeostasis^17^,^18^,^19^,^20^. However, 3D cell-centred agent-based models oversimplify keratinocyte morphology with spheres – an approximation that falls apart for cells in the stratum granulosum (SG) and particularly for the stratum corneum (SC): Here, cells typically measure 30 μm in length and width, but less than 1 μm in height^10^. Simple geometry precludes correctly recreating SC morphology and thickness with spherical cells. Additionally, most models fail to address skin hydration and the barrier function of the SG and SC. Notably, the irregular cell shape of SG and SC cells also affects the barrier function^1^,^21^. In terms of modelling, an irregular cell shape impacts the number of direct neighbours when cells in close proximity have vastly different shapes. Furthermore, irregular shapes affect biophysical properties and biochemical reactions that depend on surface area or volume, e.g. cell-cell adhesion proportional to the density of adhesive proteins or production of signalling molecules by cytosolic enzymes.

Here, we present the first 3D self-organizing epidermis model that combines realistic and dynamic cell morphology with barrier formation sensitive to environmental cues. Our model includes calcium transport, water flow and ambient humidity. The regulatory interplay between the epidermal barrier and these transport processes allows the *in silico* epidermis to self-organize into stratified layers displaying calcium and water gradients comparable to *in vivo* data. To allow a dynamic cell shape, we developed a 3D off-lattice cell-centre-based biomechanical model (BM), thus extending the BM portfolio of the modelling and simulation platform EPISIM^18^,^22^. This platform uniquely allows creating agent-based models intuitively through a graphical modelling language, enabling less computer-savvy users to create complex cell behavioural models (CBM).

The new BM yields a SC with realistic flattened corneocyte morphology. In our new CBM, cells in the SG and SC flatten and produce tight junctions and lipids that impede diffusion, thus generating an epidermal barrier. Other model elements such as the cell cycle and differentiation model have been adapted from earlier published work^18^,^22^. We use the model to simulate *in silico* growth from a small number of stem cells seeded on a substrate to a full-grown 3D homeostatic epidermis with thousands of cells. We perturb our *in silico* epidermis by altering environmental humidity, and show that it robustly returns to homeostasis after a transient adaptation period. This comprehensive model of homeostatic epidermis provides a stepping stone for future work to investigate how dysregulation of homeostasis leads to skin disease.

## Results

### Biomechanical interactions of ellipsoid cells lead to realistic epidermal morphology

We developed a model of epidermal homeostasis that includes a realistic cell morphology model based on ellipsoids as well as biomechanical forces between cells, and the interplay of the epidermal barrier with calcium and water balance in the tissue. For biomechanics, we developed a cell-centre-based model that incorporates ellipsoid cell shapes. To this end, we implemented an approximation to calculate the surface of contact between ellipsoids, which we use to scale adhesive forces (for a comprehensive description, the reader is referred to the Supplementary Information).

**Figure 1:**
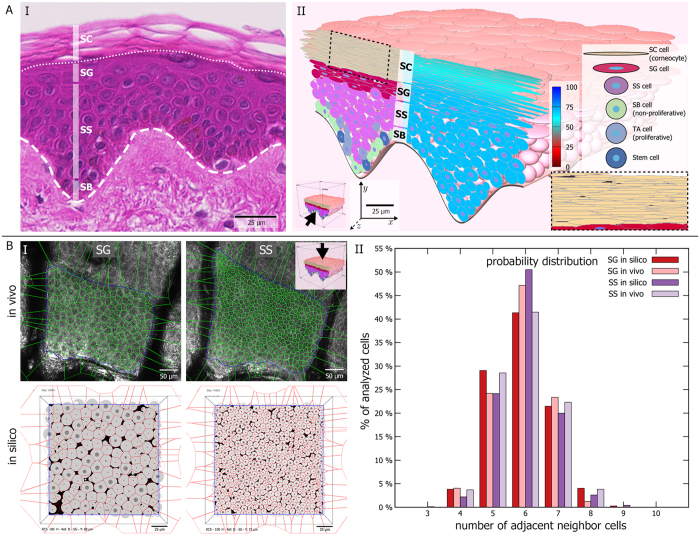
The biomechanical model of ellipsoid cells allows to faithfully recreate epidermal morphology. (**A**) (I) Using ellipsoid cells, we can attain realistic tissue morphology as observed in histological preparations, including the typical epidermal layering consisting of stratum basale (SB), stratum spinosum (SS), stratum granulosum (SG), and stratum corneum (SC). (II) Our biomechanical model leads to the typical interdigitating SC pattern (inset). The left half of the 3D tissue cross-section shows colouring according to cell differentiation status. The right half displays cellular water content. Inner blue circles represent cell centres (not shown for SC due to the narrow cell size). (**B**) (I) Topological analysis of SG and SS based on manually segmented cells in *in vivo* images and cross-sectional images of the *in silico* epidermis. (II) The probability distribution of nearest neighbours per cell *in silico* follows closely the *in vivo* distribution.

In steady state, the *in silico* epidermis consists of four cell layers (Fig. 1A). Stem cells and transit amplifying cells in the stratum basale (SB) proliferate to generate keratinocytes. As previously described^18^,^22^, stem cells in the model proliferate asymmetrically to generate one stem cell and one TA cell daughter. TA cells divide asymmetrically up to three times, generating one TA (with proliferative potential diminished by one) and one non-proliferative SB cell. After three divisions, TA cells become non-proliferative SB cells. Upon losing contact with the basement membrane, TA cells and non-proliferative SB cells differentiate to stratum spinosum (SS) cells. Upon attaining either high calcium content or low water content, these SS cells differentiate to stratum granulosum (SG) cells. In turn, SG cells mature into corneocytes that build up the stratum corneum (SC); sustained low water content triggers immediate differentiation of SG into SC cells to simulate cell desiccation (see Supplementary Fig. S3). At each differentiation step, the cells flatten to a shape characteristic of their new differentiation status and their adhesive properties change. The interplay between repulsive and adhesive forces leads to equilibration of cells to optimally distribute in space and form the typical layered structure of the epidermis. Cell turnover and differentiation continuously perturb this equilibrium – nevertheless the overall steady state epidermal morphology remains constant (see Supplementary Video S1).

Adhesive forces determine the desquamation rate of SC cells in the model. Corneocytes in the model initially increase their adhesion mainly at the narrow lateral contacts. In a second phase, adhesive strength decreases progressively to simulate enzymatic degradation of cellular bonds (see Supplementary Eq. 29–30 and accompanying text). We base both of these rules on experimental observations^23^,^24^. Surface corneocytes desquamate when their average adhesive strength to all neighbours falls under a threshold. Using these rules, we obtain the characteristic pattern of corneocyte interdigitation (Fig. 1A–II, inset). Combining differentiation with flattening of cells to an ellipsoid shape allows our model to closely correspond to histological morphology (Fig. 1A). Further, topological analysis of experimental data acquired by *in vivo* confocal reflectance microscopy^25^ matches well to the neighbour topology that emerges from the interactions of biomechanics with cell turnover and differentiation in our model (Fig. 1B).

### The *in silico* epidermal tissue self-organizes from feedback loops

**Figure 2:**
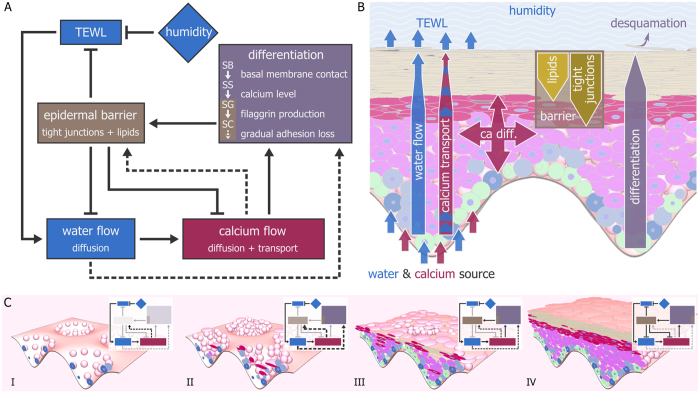
Cells in the model self-organize into a layered epidermis through several regulatory feedback loops. (**A**) The core regulatory feedback loops underlying the model. The global parameter humidity (H) inversely affects trans-epidermal water loss (TEWL), which directly affects water flow and indirectly calcium flow (solid arrows). Without the epidermal barrier, water and calcium levels decrease, triggering non-homeostatic differentiation and barrier formation (dashed arrows). The epidermal barrier locally slows down TEWL, water flow, and calcium flow, allowing calcium to accumulate in the SS to trigger homeostatic differentiation (solid arrows). Differentiated cells in the SG and SC build up the barrier in homeostasis. The negative feedback loop between calcium flow and the epidermal barrier ensures a constant homeostatic thickness of the tissue. (**B**) Schematic representation showing the spatial organization of the barrier, water, and calcium flow in homeostasis. Cells in contact with the basal membrane have access to a source of water and calcium. Due to TEWL, surface cells lose water, which triggers apical water flow by diffusion and calcium flow by directed transport coupled to the water flow. Calcium transport is counteracted by diffusion. Water and calcium flow are locally reduced by the barrier, which consists of stacks of flattened SG and SC cells containing tight junctions (SG and SC) or lipids (SC). The barrier leads to local calcium accumulation in the underlying cells. (**C**) Different regulatory loops become active as the model epidermis grows from a few stem cells (I) to a full homeostatic tissue (IV). Initially, the epidermal barrier is absent, leading to uncontrolled water and calcium flow (I). This activates the non-homeostatic rescue mechanisms (dashed arrows), which build a transient ectopic barrier (II and III). This transient barrier shields the underlying layers, which can differentiate normally (IV).

The epidermis evolved as a barrier between the organism and the outside environment, and primarily protects against water loss. In our model, we model how cellular feedback loops involving stimuli external and internal to the tissue generate and maintain this barrier function (Fig. 2A). In the following, we delineate the main components of the model, and briefly describe the role these components play as the simulated tissue grows. The flow of water and calcium is a central component of our model (see Supplementary Fig. S3C). We assume that all epidermal water flow occurs passively by diffusion (see Supplementary Eq. 31–34), whereas calcium flow occurs both by diffusion and by transport coupled to the water flow (see Supplementary Eq. 35–42). The latter may be interpreted as simple advection or as an actively regulated transport process. Additionally, since water and calcium content are cell properties, our cell-based approach intrinsically incorporates convection due to cell movement. Since we follow a strictly cell-based approach, we implemented diffusion and other transport processes as occurring between a cell and its direct neighbours (see Supplementary Fig. S4B; Supplementary. Eq. 33; 35–37). By simulating molecular transport from cell to cell, transport takes longer through a stack of flat cells compared to an equally tall pile of round cells. Thus, this abstraction entails a simple representation of tortuosity – a known property of flattened SG and SC cells that contributes to the barrier function^7^,^8^. We model only cells in the epidermis, but implicitly we include the effect of the underlying dermis and the external environment as boundaries. We assume that the dermis contains a fixed water and calcium content; water and calcium can flow from the dermis into epidermal cells in contact with the basement membrane (see Supplementary Eq. 34; 40–42). Further, we assume that cells at the tissue surface lose water to the external environment through evaporation (trans-epidermal water loss; TEWL), which is inversely proportional to the humidity H, a global parameter with values in the interval [0, 100] in an arbitrary scale (see Supplementary Eq. 31–32). Thus, the dermis and external environment boundaries of the tissue essentially act as a source and sink, respectively. We assume calcium can be lost in two ways. First, as water evaporates in surface cells, some calcium is lost as insoluble deposits. Second, corneocytes bind calcium, reducing the mobile pool of calcium. We use high calcium content as a signal for homeostatic differentiation from SS to SG, based on experimental observations^12^,^13^,^26^.

In our model, the flow of water and calcium can be locally damped by the epidermal barrier (see Supplementary Eq. 43–46). *In vivo*, epidermal cells have homeostatic and non-homeostatic pathways to create the epidermal barrier^27^. In the model, the barrier consists of tight junctions and lipids, which cells gradually produce during their lifetime in the homeostatic pathway (see Fig. 2A,B, solid arrows; Supplementary Fig. 3D). We model the non-homeostatic pathway as follows: Cells in SS and SG react to low calcium by prematurely producing tight junctions and lipids, whereas sustained low water levels lead to premature differentiation and ultimately desiccation (see Fig. 2A, dashed arrows; Supplementary Fig. 3E,F). Prematurely differentiated SG cells behave as their homeostatic counterparts, and thus can actively contribute to barrier formation. In contrast, prematurely differentiated SC cells are considered dead and merely act as a passive obstacle; they produce no lipids. We base these rules on experimental observations^28^,^29^,^30^.

The aforementioned processes lead to a self-regulatory epidermal tissue. The simulated epidermal tissue, including cell differentiation and layer formation, develops in two phases: First, a barrier-generating phase, during which the epidermis grows from a small number of initial stem cells, and second, a homeostatic barrier-maintenance phase. Importantly, the switch from one phase to the next occurs based on local cellular decisions. Thus, our model entails a wholly self-regulatory epidermis. At the start of a simulation, the epidermis consists solely of stem cells seeded on the undulated basement membrane of the rete ridges and completely lacks a barrier (Fig. 2C–I). Our starting condition is inspired by *in vitro* epidermal tissue culture. Due to TEWL, water tends to deplete in surface cells, leading to a passive water flow from basal to apical cells (Fig. 2A,B; see also Supplementary Video S2). In turn, this leads to upward transport of calcium, which is counteracted by calcium diffusion (Fig. 2B). However, in the absence of a barrier, surface cells lose water and calcium at a rate inversely proportional to H. Therefore, SS cells cannot differentiate into SG through the homeostatic pathway (Fig. 2A, solid arrows). In this regime, non-homeostatic pathways activate and create an initial barrier constituted of tight junction and lipid-carrying SS cells as well as prematurely differentiated SG and SC cells (see Fig. 2C–II,III; see Supplementary Fig. S3E,F). This initial barrier reduces water and calcium loss, allowing enough calcium to accumulate for homeostatic differentiation of the next generations of SS cells into SG cells (Fig. 2C–III). In summary, the non-homeostatic feedback mechanisms can establish an initial barrier (Fig. 2C–II,III), which lets the tissue achieve homeostasis (Fig. 2C–IV). Once in homeostasis, the barrier locally reduces the flow of water and calcium, maintaining a calcium gradient with peak in the upper SS and SG (see also Supplementary Videos S2–S3). The calcium gradient peak ensures continuous differentiation of SS cells into SG cells, which in turn produce the barrier. Thus, once a barrier is present, the tissue self-perpetuates its homeostasis.

### Growing the tissue at low humidity extends the pre-homeostatic phase

**Figure 3:**
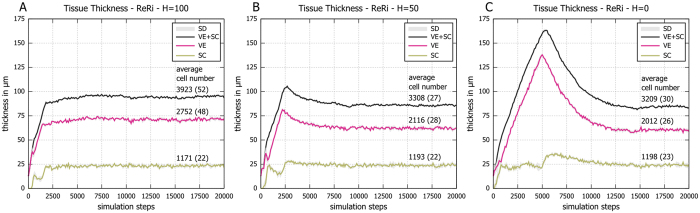
The *in silico* epidermal tissue overshoots its ideal thickness at low humidity. (**A**) At high H, both the viable epidermis (VE) and the stratum corneum (SC) rapidly reach their steady state thickness. (**B**) Lowering H leads to a longer period of equilibration accompanied by a small overshoot in VE thickness. (**C**) Further lowering H leads to a dramatic overshoot in VE thickness, but comparably little effect on the SC. The thickness of VE and SC was measured over time in 6 subvolumes of one simulation run for each condition (see Supplementary Fig. S5). Shown are averaged values + standard deviation (SD) for the deepest part of the rete ridges. Average cell numbers are given for the simulated epidermis between simulation step 15,000 to 20,000; SD in parentheses.

**Figure 4:**
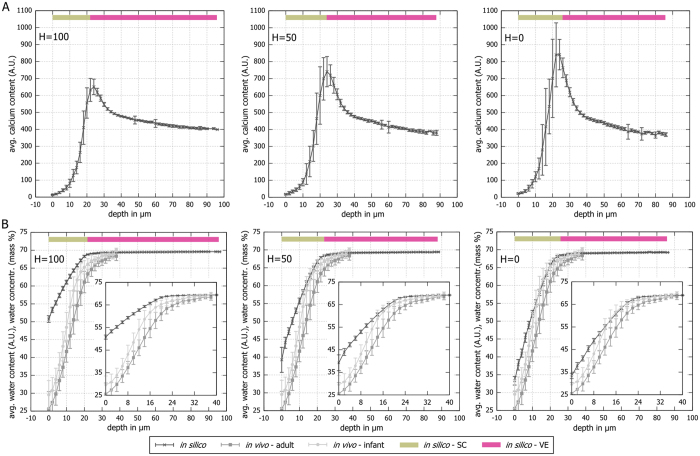
Steady state water and calcium gradients steepen with decreasing humidity. (**A**) *In silico* calcium profile as a function of depth at different values of H; 0 μm is defined as the surface of the tissue. The calcium gradient steepens as humidity decreases. Regardless of condition, the calcium content peaks in the border between viable epidermis and SC. (**B**) *In silico* water profiles as function of depth at different values of H, overlaid with *in vivo* hydration profiles for adults and infants (used with permission from Nikolovski *et al*.^57^); 0 μm is defined as the surface of the tissue. The *in silico* epidermis at minimum H resembles most closely the *in vivo* data. Please note that the *in vivo* data is the same in all three subpanels. The *in silico* data is given in water content 0–100 (A.U.). The *in vivo* data corresponds to water concentration in percent of mass. The inset charts show a magnified picture of the SC data. Each panel represents data from one simulation in homeostasis where calcium or water content of cells at similar depths were binned for one simulation step; error bars denote standard deviation.

How does altering humidity affect the growth of the model epidermis? To answer this question, we simulated tissue growth at H = 100 (high), H = 50 (medium), and H = 0 (low), keeping all other parameters equal. The tissue achieves its homeostatic thickness very rapidly at high H, and takes progressively longer to do so at medium and low H (Fig. 3). This delay accompanies a striking overshoot in the ideal thickness of the tissue at low H, followed by a wave of cornification that briefly thickens the SC (Fig. 3C). Additionally, the steady state thickness of the tissue decreases by up to 10 μm at low H. The difference in thickness results from a steeper calcium gradient at lower H (Fig. 4A). Since high calcium content leads to SS differentiation into SG, the viable epidermis becomes thinner at low H (see Fig. 3C). The thickness of the SC remains unaffected, as it depends primarily on biomechanical adhesion parameters. Once in steady state, the *in silico* epidermis stably maintains the total number of cells and the tissue thickness (Fig. 3). Due to the uneven anatomy of the basement membrane, thickness measurements of the epidermis vary depending on location, particularly for the viable epidermis. However, at any particular location, the homeostatic thickness *in silico* remains stable (Fig. 3 shows measurements at the deepest points of the tissue; Supplementary Fig. S5 at the shallowest points). Accounting for this anatomical variance, the ranges obtained *in silico* for homeostatic viable epidermis (22–72 μm) and SC (23–28 μm) overlap well with the ranges measured experimentally in the arm region: 30–70 μm and 10–30 μm, respectively^29^,^31^,^32^,^33^. Simulations on a flat basement membrane display qualitatively similar behaviour with respect to the impact of humidity; the homeostatic thickness is comparable to tissue thickness in shallow points of tissue simulated on the undulated basement membrane (see Supplementary Fig. S9A–C). Interestingly, *in vitro* tissue culture systems^34^ with a flat membrane show a comparable overall thickness to our simulations on a flat membrane^35^.

All changes induced by H derive from an increased water concentration gradient between the epidermis and the external environment. Due to increased TEWL at medium and particularly at low H, many cells in the SS start prematurely differentiating and producing barrier components. This ectopic barrier protects the growing tissue (see also Fig. 2C–III,IV), but also excessively dampens water and calcium flow, leading to delayed differentiation of the layers underneath and thus to transiently increased tissue thickness. If the effect of tight junctions is removed from the model (parameters 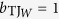, 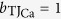; see Supplementary Eqs 43,44,46), the tissue is never able to reach homeostasis, regardless of the level of H. Similarly, if lipids are removed (parameters 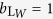, 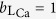; see Supplementary Eqs 43–46), tissue homeostasis only occurs at H = 100, with initial tissue thickness dynamics reminiscent of H = 0 in simulations with unchanged parameters (see Supplementary Fig. S11). In both cases we see an emergent compensatory thickness overshoot behaviour, indicating that the lipid-less barrier at H = 100 dampens water flow in a similar way as the full barrier at H = 0. Thus, tight junctions contribute to a greater extent to formation of an initial barrier in the model, but both tight junctions and lipids are needed to robustly attain homeostasis at different environmental conditions.

### The epidermal barrier buffers the effects of humidity on the viable epidermis

Regardless of the value of H, the virtual epidermis eventually settles into a steady state exhibiting the typical shapes for calcium and water gradients along tissue depth (see Fig. 4, Supplementary Fig. S6). The water flow drives calcium flow from the SB to the SC, where it is bound by corneocytes (thus removing calcium from the mobile pool). As a result, calcium increases in the viable epidermis (SB, SS, and SG), peaks at the border to the SC, and then declines sharply (Fig. 4A). The barrier prevents diffusive dissipation of the peak of epidermal calcium by locally reducing water and calcium flow to different degrees, *i.e.* water flow-coupled calcium transport has a stronger contribution than calcium diffusion. At the same time, the barrier also ensures that decreasing H steepens water profiles only in the upper SG and SC (Fig. 4A,B). Comparing the *in silico* water profile to *in vivo* data obtained from adults and children suggests that the simulation at low H most closely matches the *in vivo* condition (Fig. 4B).

**Figure 5:**
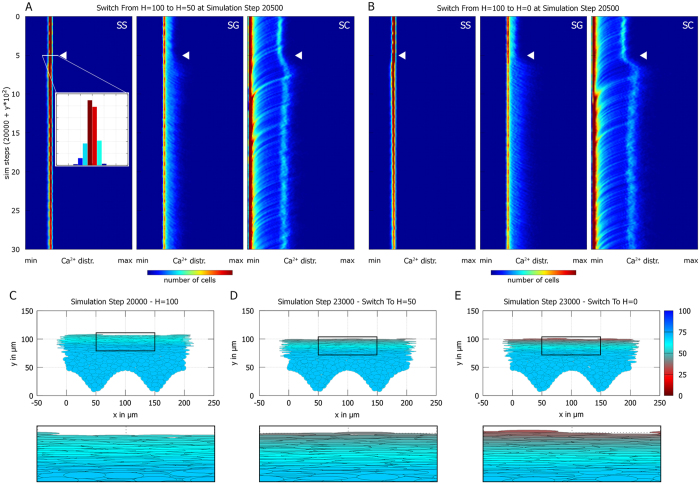
The epidermal barrier in the stratum corneum buffers changes in humidity. **(A,B)** 2D histograms over time of the calcium distribution in SS, SG, and SS. Each horizontal line corresponds to the distribution at a single timepoint; the color code indicates the total number of cells. (**A**) The simulation started at H = 100 and was switched to H = 50 after attaining homeostasis (white arrowhead). The calcium distribution in the SS barely changes; in the SG cells tend to accumulate higher levels of calcium, leading to a shift to higher levels in the SC. **(B)** Simulation with a switch from H = 100 to H = 0 (white arrowhead). **(C–E)** Water content in simulated tissue at H = 100 (**C**), after a switch from H = 100 to H = 50 (**D**), and after a switch from H = 100 to H = 0 (**E**). The effects of changing H on the water profile remain restricted to the SC (insets). Refer online to Supplementary Fig. S7 for a control simulation where H was not altered.

Next, we tested how changing H during a simulation affects the tissue after it has achieved homeostasis at H = 100 with a mature barrier (see Fig. 5, Supplementary Fig. S7 and Supplementary Video S4). As before, reducing H barely affects the SS, but leads to steeper calcium and water gradients in SG and SC. When plotted in a 2D histogram of calcium content by cell type over time, this manifests as an increase in the maximum calcium content in the SG and particularly in the SC (Fig. 5A,B). The bimodality of the calcium distribution in SG and SC arises from cells at different depths in the tissue having different calcium content: The maximum calcium content is attained in a narrow band of cells at the border of SG and SC (compare Fig. 4A). Thus, recently differentiated corneocytes close to the viable epidermis have high calcium (right peak in the 2D histogram in Fig. 5A,B, right panel). Over time, individual corneocytes bind calcium and thus reduce their calcium content, leading to diagonal streaks moving from right (high calcium) to left (low calcium) in the 2D histogram (Fig. 5A,B, right panel). Instead, individual SG cells accumulate calcium over time, which leads to diagonal streaks moving from left (low calcium) to right (high calcium; Fig. 5A,B, middle panel).

The epidermal barrier mitigates the impact of H on tissue homeostasis: When the *in silico* epidermis grows from scratch at low H, it takes up to 10000 simulation steps to achieve homeostasis (Fig. 3C). In contrast, when we reduce H only after the tissue developed a mature barrier, the equilibration phase occurs in less than 500 simulation steps (Fig. 5B). Furthermore, water and calcium distribution in the SS remain almost unchanged (Fig. 5A,B). Finally, we performed *in silico* tape stripping experiments to test how the homeostatic epidermis reacts to removal of the barrier. *In vivo*, tape stripping involves invasive removal of the epidermal barrier; the procedure aims to remove the entire SC, but can also affect the SG^24^. The *in silico* epidermis reacts to tape stripping of the SC with a small transient change in homeostatic thickness, indicating that the SG by itself already contributes greatly to the epidermal barrier strength in the model (see Supplementary Fig. S10A). Indeed, removal of both the SG and the SC has a more striking effect, as the tissue overshoots its ideal thickness before returning to the steady state (see Supplementary Fig. S10B). This thickness overshoot resembles the effect seen when letting the *in silico* epidermis grow from stem cells (compare Fig. 3A). Interestingly, a thickness overshoot of the viable epidermis has also been observed in tape stripping experiments *in vivo*^29^,^36^. Summarizing, the model epidermis grows best at high H, but once it attains homeostasis it reacts robustly to changes in H, underscoring the importance of the epidermal barrier in maintaining epidermal health.

## Discussion

### The model replicates key aspects of epidermal homeostasis, barrier, and hydration

The epidermis lies at the interface of two worlds, acting as mediator between the external environment and the internal homeostasis of the organism. In terrestrial animals, its most important function consists of preventing dehydration by stemming evaporation – a function performed chiefly by the stratum corneum (SC). In this work, we expanded our previously published multicellular model of epidermis^18^,^22^ to incorporate the SC. To accommodate the extremely flattened shape of SC cells, we developed a new biomechanical model. Moreover, we focused on modelling the interplay of skin barrier strength, ambient humidity, and epidermal water and calcium gradients – all of which are generally considered important parameters for skin health^1^,^37^. Low environmental humidity leads to a more resilient barrier^38^, whereas loss of the epidermal calcium content is seen as a wounding signal and leads to precipitous barrier reorganization^28^.

To reflect this, we incorporated feedback mechanisms that allow cells to quickly rebuild the epidermal barrier when they experience low water or low calcium content. All these elements together allow the model to replicate the experimental observations summarized in Table 1. Despite using only qualitative parameters, we obtain a decent quantitative match to *in vivo* water profiles.

### New biomechanical model replicates realistic epidermal morphology

To realistically depict corneocyte morphology, we developed a 3D off-lattice cell-centre-based BM that allows using ellipsoid cell shapes. Other types of models such as subcellular element models entail a more refined approach, potentially able to represent any arbitrary cell shape^39^. Such models have begun to be used to model how stratification first occurs in the epidermis during development^40^,^41^. However, introducing multiple subcellular elements for each cell comes at a computational cost. Therefore, we opted for the reasonably realistic compromise of a cell-centre-based ellipsoid shape.

To our knowledge, there is only one other example of a 3D cell centre agent-based model using ellipsoid cells, in a model developed by Dallon & Othmer^42^, but cells in this model deviated only little from perfect spheres, permitting use of a rough approximation for the contact area between two cells. When used to calculate contact areas of extremely irregular ellipsoids, this approximation overestimates contact at narrow edges, and underestimates contact at broad and flat edges (see Supplementary Information for an in-depth analysis). To overcome this limitation in our BM, we incorporate a more accurate approximation. The new BM still retains use of the approximation by Dallon & Othmer, as it provides fast and sufficiently accurate solutions for nearly spherical cells. To minimize computational effort, we use the new approximation only when strictly required by the model, *e.g.* for transport of water and calcium.

Counter to intuition, human corneocytes adhere strongly at their narrow lateral contacts and have relatively poor adhesion at their large apico-basal contact surface^23^. To reflect this, we take advantage of the lateral overestimation and apico-basal underestimation given by the Dallon & Othmer approximation to calculate corneocyte cell adhesion forces. Simulating the epidermis with a strong lateral adhesion of corneocytes results in the typical pattern of corneocyte interdigitation. In contrast, if adhesion strength is kept proportional to contact area using our more accurate approximation, corneocytes stack into columns (see Supplementary Fig. S12). Interestingly, this column-like morphology is reminiscent of the SC structure in mouse epidermis^43^, suggesting that mouse SC has different adhesion properties to human SC. This may be due to the water-retaining properties of the fur coat, which reduces the need for a strong epidermal barrier^44^,^45^.

In the simulations presented here, we focused on attaining a realistic steady state morphology of the tissue. In particular, we restricted ourselves to feedback interactions occurring at the level of differentiated cells, leaving the proliferative compartment static. The initial condition with stem cells seeded on a substrate has been inspired by *in vitro* tissue culture systems. A computational study of the proliferative compartment of developing embryonic epidermis identified predominant stem cell asymmetric divisions and polarized adhesion protein distribution at the basement membrane as necessary for orderly stratification^41^. Interestingly, the cell proliferation model that we adapted from previous work makes similar assumptions^18^,^22^. Thus, despite the different approach and simplified cell proliferation model, our study captures the essential qualities required of the proliferative compartment.

### Computational models – an asset for quantitative skin research

The traditionally strong experimental focus in biology prevents the more widespread application of computational models in skin research. Thus, computational modelling must, first, become more accessible to the general scientific community, and second, must be able to recreate a reasonably realistic representation of skin. With the first goal in mind, we have previously introduced EPISIM, a platform for graphical multi-scale modelling and cell-based simulation of multicellular systems^18^,^22^. In this work we placed the focus on the second goal, *i.e.* producing a model of epidermis that summarizes its most important functions while maintaining a realistic morphology.

Computational models rely on data, but can also be used to extrapolate from existing knowledge^46^. An engineer attempting to replicate M. C. Escher’s structures would quickly realize their contradiction to our physical reality. Similarly, assembling our current understanding of a biological system *in silico* intrinsically checks the consistency of the underlying biological hypotheses. One prominent hypothesis in epidermal research entails the TEWL-driven emergence of the calcium gradient^47^,^48^. In this regard, our model makes an important contribution as it points us to the importance of the balance between calcium diffusion and TEWL-directed calcium transport. When diffusion trumps directed transport, the gradient dissipates; this particularly affects the calcium peak next to the extremely low SC calcium content. Our model shows that the gradient can only persist when the epidermal barrier locally restricts both calcium diffusion and transport in the SG and SS. Analogous results were found in mathematical continuum models investigating the calcium gradient in the epidermis^49^,^50^. Thus, computational modelling highlights that TEWL alone cannot account for the calcium gradient unless coupled to the epidermal barrier.

### When homeostasis fails: Disease emergence

Understanding healthy skin homeostasis is a necessary first step to understand its disease-causing dysregulation. Healthy skin maintains its homeostasis when challenged by external insults. In contrast, in the disease state, symptom severity varies with temperature and ambient humidity *e.g.* for inflammatory skin dermatoses^38^,^51^; symptoms may even temporarily subside leading to apparently healthy skin. In abstract terms, these skin conditions represent a non-robust state that loses homeostasis upon perturbation.

In a computational model, we can interrogate which parameters can be manipulated to shift a non-robust homeostatic state to a robust state, thus identifying more precisely which processes need to be manipulated in this context. However, a necessary first step to attain this goal is a good model of homeostasis. Hence, we focused our efforts on the homeostatic skin state and presented the first 3D epidermis model which combines realistic cell morphology with a selection of important skin parameters such as the epidermal barrier, skin hydration, and calcium content. Despite being a proof-of-principle model using only qualitative parameters, our *in silico* epidermis presents characteristics compatible with experimental observations and responds robustly to changes in environmental cues. Thus, our comprehensive model of homeostatic epidermis provides a stepping stone for future work to investigate how dysregulation of homeostasis leads to skin disease.

## Methods

### EPISIM multiscale multicellular modelling and simulation platform

We previously introduced the EPISIM platform for graphical multi-scale modelling and cell-based simulation of multicellular systems^18^,^22^. The EPISIM platform consists of two ready-to-use software tools: (i.) EPISIM Modeller (graphical modelling system) and (ii.) EPISIM Simulator (agent-based simulation environment). Each EPISIM-based model is composed of at least a cell behavioural and a biomechanical model (CBM and BM). The BM covers all spatial and biophysical cell properties; the EPISIM simulation environment offers a multitude of different BMs (*e.g.* lattice and off-lattice). In this work, we extend the portfolio of available BMs by introducing a novel off-lattice cell-centre-based cell morphology model with a more realistic representation of biomechanical forces. BMs can be dynamically linked to a CBM. EPISIM Modeller allows to graphically construct CBMs, *i.e.* models of cellular decisions, through process diagrams. These can be automatically translated into executable code, which is loaded in EPISIM Simulator to simulate an agent-based tissue.

### Simulation Setup

We coupled the 3D biomechanical cell morphology model to the extended CBM of human keratinocytes built in EPISIM Modeller. We then used the EPISIM platform’s built-in functions to automatically translate the CBM into executable code, which we used to perform simulations in EPISIM Simulator. We started all simulations with identical initial conditions entailing a fixed number of stem cells equidistantly seeded in the undulated rete ridges of the basement membrane. Unless otherwise noted, the growth of the *in silico* epidermis was simulated for 20,000 simulation steps with a time step of 30 minutes per simulation step. At simulation step 20,000, simulation states were saved in a snapshot; snapshots can be loaded in EPISIM Simulator to completely restore the saved state, restart the simulation at a later time point, or extract data for *post hoc* analysis. To examine the effect of changing the ambient humidity (H) after a tissue simulation has reached homeostasis, we first simulated the tissue until 20,000 simulation steps at H = 100 to obtain a homeostatic *in silico* epidermis. We then stored the state of the simulation in a tissue simulation snapshot, and loaded this snapshot to be used as starting point for new simulation runs. After relaunching the simulation, we first continued to simulate with H = 100 for 500 simulation steps to demonstrate that relaunching a stored multi-agent-based simulation has no bearing on tissue homeostasis. Then, we switched the parameter to H = 50 or H = 0, and continued simulating for 2500 simulation steps (see Supplementary Fig. S7 for a simulation run where we maintained H = 100 as a control).

The different simulation setups were run in parallel, each on an individual computer cluster node with an Intel Nehalem E5520 CPU, 2.26 GHz, 16 physical cores, 12 GB RAM and 16 parallel simulation threads. The computation time for one simulation run was between 77 and 84 hours for 20,000 simulation steps (depending on the cell number).

### Geometrical and topological analysis of *in vivo* and *in silico* cross sectional images

As previously described, we used Voronoi diagrams and Delaunay triangulation to evaluate the spatial organization of cells in relation to their neighbours in epidermal optical sections obtained by *in vivo* confocal reflectance microscopy^25^. Briefly, we calculate the Voronoi diagram and its dual Delaunay triangulation graph on a pre-selected Region of Interest (ROI) in each image (Fig. 1B). This network of polygons represents the spatial organization of the cells in the selected ROI, which we use to calculate the number of Delaunay neighbours per cell^25^.

To obtain comparable data from simulations, the *in silico* epidermis model was simulated for 20,000 simulation steps in six individual simulation runs. For each simulation run, we generated cross sections parallel to the epidermal surface with EPISIM Simulator’s built-in tissue cross section rendering engine. The cross-sections (Fig. 1B) were generated with EPISIM Simulator version 1.5.1. We arbitrarily chose two planes such that cells in the cross section were either all spinous or all granular cells. In total 12 cross sectional planes of the SG and another 12 for the stratum spinosum (SS) have been processed using the same method as for the experimental data. The entire rectangular virtual cross section was considered as a ROI for the calculation of the topological parameters.

## Additional Information

**How to cite this article**: Sütterlin, T. *et al*. A 3D self-organizing multicellular epidermis model of barrier formation and hydration with realistic cell morphology based on EPISIM. *Sci. Rep.* **7**, 43472; doi: 10.1038/srep43472 (2017).

**Publisher's note:** Springer Nature remains neutral with regard to jurisdictional claims in published maps and institutional affiliations.

## Supplementary Material

Supplementary Information File

Supplementary Video S1

Supplementary Video S2

Supplementary Video S3

Supplementary Video S4

## Figures and Tables

**Table 1:** Experimental findings replicated by the 3D *in silico* epidermis model.

Experimental finding	Model characteristic	References
Epidermis responds to barrier injury by transient thickening and wave of cornification	Simulations starting at high TEWL (equivalent to injured barrier) transiently overshoot epidermal thickness. Epidermis builds up transient barrier of desiccated cells (Fig. 3). Simulated tape stripping either by removing all SC and SG cells leads to transient overshoot of epidermis (see Supplementary Fig. S10).	29, 36, 38 and 52
(i.) Skin tissue culture initially is kept submerged to allow stratification and (ii.) skin regeneration is boosted by occlusion	Tissue growth and stratification from seeded stem cells optimal at high humidity (Fig. 3)	53 and 54
Calcium gradient steepness correlates with TEWL	Low humidity (equivalent to high TEWL) results in a steeper calcium gradient (Fig. 4)	47, 48, 52 and 55
Wetting skin increases stratum corneum (SC) water content	High humidity results in greater SC water content (Fig. 4)	56
