# Supplementary material for: A 3D self-organizing multicellular epidermis model of barrier formation and hydration with realistic cell morphology based on EPISIM
Source: Sci Rep. 2017 Mar 6;7:43472. doi: 10.1038/srep43472 (PMC5338006; doi:10.1038/srep43472)
Supplement: Supplementary Information File [file srep43472-s1.pdf]

Supplementary Information

A 3D self-organizing multicellular epidermis model of barrier formation and hydration with realistic cell morphology based on EPISIM

Thomas Sütterlin[1,2,+], Erika Tsingos[1,3,+], Jalil Bensaci[4], Georgios Stamatas[4], and Niels Grabe[1,2,*]

[1]Hamamatsu TIGA Center, BioQuant, Heidelberg University, Im Neuenheimer Feld 267, 69120 Heidelberg, Germany

[2]National Center for Tumor Diseases, Dept. of Medical Oncology, Im Neuenheimer Feld 460, 69120 Heidelberg, Germany

[3]Centre for Organismal Studies, Heidelberg University, Im Neuenheimer Feld 230, 69120 Heidelberg

[4]Emerging Science & Innovation, Johnson & Johnson, Santé Beauté France, 1 rue Camille Desmoulins, 92130 Issy les Moulineaux, France

[*]corresponding author: niels.grabe@bioquant.uni-heidelberg.de

[+]these authors contributed equally to this work.

Centre-based biomechanical cell morphology model

Cell morphology model

Cell centre-based models allow cells to move freely in space and consider all forces as acting on a cell's centre of mass[1,2]. Usually, a cell's spatial representation is either a circle or a sphere[3–8]. Only few centre-based models use ellipses or ellipsoids as spatial representation for cells, but none of these considers cells with highly irregular shape[9,10]. Our biomechanical cell morphology model is an off-lattice, centre-based model designed to reflect a flattened cell morphology. Hence, cells are modelled with ellipsoids to account for irregular keratinocyte morphology and especially the flattened corneocyte morphology. A cell ellipsoid with semi-principal axes a, b and c is given by:

$$E \equiv (\mathbf{x}-\mathbf{r})^{\mathrm{T}} M^2 (\mathbf{x}-\mathbf{r}) = 1 \tag{1}$$

$$\text{with} \quad M = \begin{pmatrix} \frac{1}{a} & 0 & 0 \\ 0 & \frac{1}{b} & 0 \\ 0 & 0 & \frac{1}{c} \end{pmatrix}$$

The ellipsoids have 3 degrees of freedom such that orientation changes based on intercellular forces are neglected, a simplification also used in other centre-based models using ellipses or ellipsoids as spatial representation for cells[9,10]. The axes a, b, c are used in the cell morphology model to control the width ($2a$), height ($2b$), and length of cells ($2c$). Supplementary Table S1 lists the keratinocyte sizes used in our model for each differentiation stage. The projected cell areas reported in literature suggest that cells have always the same length and width [11]. Thus, we chose to set $a = c$.

keratinocyte	semi-major axis *a* and *c*	semi-minor axis *b*	Reference
corneocytes	17 µm	0.75 µm	11
granular cells	12 µm	2 µm	derived from 11,12
spinous cells	6 µm	4 µm	12
basal cells	5 µm	5 µm	12

Supplementary Table S1. Keratinocyte cell sizes based on differentiation stage

Biomechanical model

Cells in a tissue simulation using our biomechanical model (BM) equilibrate the distance to each of their adjacent cells by exerting pressure forces F_{pr} or adhesion forces F_{adh}. The distance equilibrium is continuously perturbed by proliferating cells in the basal layer, whose daughter cells exert pressure on their cellular environment. Additionally, cell shape changes during differentiation and desquamation of corneocytes can lead to cell rearrangement. The target value of the force equilibration process is the optimal distance d_{opt} (Supplementary Figure S2), which is calculated in the following three steps:

1. determination of line L through cell centres (of mass) $\mathbf{r}_{\mathrm{c}}$ and $\mathbf{r}_{\mathrm{n}}$ with direction vector $\mathbf{v}_{\mathrm{cn}} = \mathbf{r}_{\mathrm{n}} - \mathbf{r}_{\mathrm{c}}$ (and inverted direction vector $\mathbf{v}_{\mathrm{nc}} = \mathbf{r}_{\mathrm{c}} - \mathbf{r}_{\mathrm{n}}$) (see Supplementary Figure S2)
2. calculation of the intersection points of line L with the ellipsoid surface
3. summation of the line segments $d_{\mathrm{seg\_cn}}$ and $d_{\mathrm{seg\_nc}}$

Consequently, the optimal distance d_{opt} between two cells located at $\mathbf{r}_{\mathrm{c}}$ and $\mathbf{r}_{\mathrm{n}}$ is:

$$d_{\mathrm{opt}}(\mathbf{r}_{\mathrm{c}}, \mathbf{r}_{\mathrm{n}}) = \underbrace{\left\| \frac{\hat{\mathbf{v}}_{\mathrm{cn}}}{\sqrt{\left(\frac{\hat{v}_{\mathrm{cn}_1}^2}{a_{\mathrm{c}}^2} + \frac{\hat{v}_{\mathrm{cn}_2}^2}{b_{\mathrm{c}}^2} + \frac{\hat{v}_{\mathrm{cn}_3}^2}{c_{\mathrm{c}}^2}\right)}} \right\|}_{d_{\mathrm{seg}_{\mathrm{cn}}}} + \underbrace{\left\| \frac{\hat{\mathbf{v}}_{\mathrm{nc}}}{\sqrt{\left(\frac{\hat{v}_{\mathrm{nc}_1}^2}{a_{\mathrm{n}}^2} + \frac{\hat{v}_{\mathrm{nc}_2}^2}{b_{\mathrm{n}}^2} + \frac{\hat{v}_{\mathrm{nc}_3}^2}{c_{\mathrm{n}}^2}\right)}} \right\|}_{d_{\mathrm{seg}_{\mathrm{nc}}}} \tag{2}$$

$$\text{with} \quad \hat{\mathbf{v}}_{\mathrm{cn}} = \frac{\mathbf{v}_{\mathrm{cn}}}{\|\mathbf{v}_{\mathrm{cn}}\|} \quad \text{and} \quad \hat{\mathbf{v}}_{\mathrm{nc}} = \frac{\mathbf{v}_{\mathrm{nc}}}{\|\mathbf{v}_{\mathrm{nc}}\|}$$

The adhesion force between a basal cell and the basement membrane depends on the optimal distance between a cell located at $\mathbf{r}_{\mathrm{c}}$ and its membrane reference point $\mathbf{r}_{\mathrm{bm}}$ which is calculated as follows:

$$d_{\mathrm{opt}}(\mathbf{r}_{\mathrm{c}}, \mathbf{r}_{\mathrm{bm}}) = \left\| \frac{\hat{\mathbf{v}}_{\mathrm{c\_bm}}}{\sqrt{\left(\frac{\hat{v}_{\mathrm{c\_bm}_1}^2}{a_{\mathrm{c}}^2} + \frac{\hat{v}_{\mathrm{c\_bm}_2}^2}{b_{\mathrm{c}}^2} + \frac{\hat{v}_{\mathrm{c\_bm}_3}^2}{c_{\mathrm{c}}^2}\right)}} \right\| \tag{3}$$

$$\text{with} \quad \hat{\mathbf{v}}_{\mathrm{c\_bm}} = \frac{(\mathbf{r}_{\mathrm{bm}} - \mathbf{r}_{\mathrm{c}})}{\|\mathbf{r}_{\mathrm{bm}} - \mathbf{r}_{\mathrm{c}}\|}$$

The membrane reference point $\mathbf{r}_{\mathrm{bm}}$ is determined for each basal cell at each simulation step and corresponds to the point on the basal membrane with minimal distance to a cell's centre $\mathbf{r}_{\mathrm{c}}$ (Supplementary Figure S1). For determining the intercellular pressure, the overlap d_{ol} of two adjacent cells has to be calculated:

$$d_{\mathrm{ol}}(\mathbf{r}_{\mathrm{c}}, \mathbf{r}_{\mathrm{n}}) = \hat{d}_{\mathrm{opt}}(\mathbf{r}_{\mathrm{c}}, \mathbf{r}_{\mathrm{n}}) - \|\mathbf{v}_{\mathrm{nc}}\| \tag{4}$$

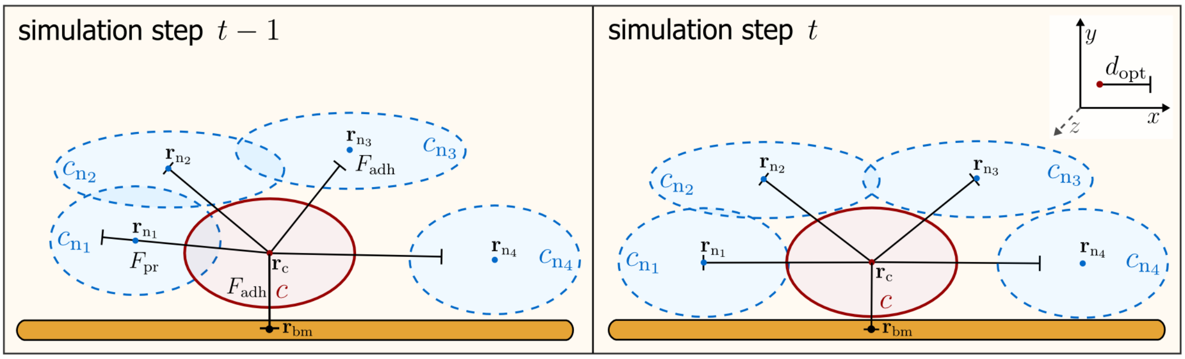

Supplementary Figure S1: New cell centre-based biomechanical cell morphology model with ellipsoidal cell morphology. Intercellular pressure force F_{pr} and cell-cell-adhesion force F_{adh} lead to an equilibration of the cell distances between a cell c and its neighbouring cells c_{n_i}. Cell locations are $\mathbf{r}_\mathrm{c}$ and $\mathbf{r}_{\mathrm{n}_i}$ respectively. Basal cells adhere to the basal membrane in with force proportional to the size of the contact area. The contact area is calculated based on the distance to a reference point $\mathbf{r}_{\mathrm{bm}}$ on the basal membrane, *i.e.* the point with the smallest distance to cell centre $\mathbf{r}_\mathrm{c}$.

$$\hat{d}_{\mathrm{opt}}(\mathbf{r}_\mathrm{c},\mathbf{r}_\mathrm{n}) = \delta_{\mathrm{ol}} d_{\mathrm{opt}}(\mathbf{r}_\mathrm{c},\mathbf{r}_\mathrm{n}) \tag{5}$$

The overlap is defined as the difference between the scaled optimal distance and the real distance. As ellipses are not space filling we set the tolerated overlap to 15% of the optimal distance ($\delta_{\mathrm{ol}} = 0.15$ see also[13]). We impose a 'hard-core' constraint on intercellular pressure to limit a cell's compressibility. To compute the maximum compression, we define a maximum cell overlap $d_{\mathrm{ol\_max}}$ corresponding to 50% of the optimal distance ($\delta_{\mathrm{ol\_max}} = 0.5$).

$$d_{\mathrm{ol\_max}} = \delta_{\mathrm{ol\_max}} \hat{d}_{\mathrm{opt}}(\mathbf{r}_\mathrm{c},\mathbf{r}_\mathrm{n}) \tag{6}$$

The intercellular pressure force $F_{\mathrm{pr}}(\mathbf{r}_\mathrm{c},\mathbf{r}_\mathrm{n})$ between two neighbouring cells located at $\mathbf{r}_\mathrm{c}$ and $\mathbf{r}_\mathrm{n}$ is the overlap multiplied with the linear spring constant k_{pr}. If the minimal relevant overlap $d_{\mathrm{ol\_min}}$ is exceeded without violating the 'hard-core' constraint, intercellular pressure increases linearly with increasing overlap. As soon as the overlap exceeds $d_{\mathrm{ol\_max}}$, the pressure increases exponentially to ensure a minimal separation of the cells. The chosen spring constant k_{pr} is $2.2 \times 10^{-3}\mathrm{N\ m}^{-1}$[14].

$$F_{\mathrm{pr}}(\mathbf{r}_\mathrm{c},\mathbf{r}_\mathrm{n}) = \begin{cases} k_{\mathrm{pr}} d_{\mathrm{ol}}(\mathbf{r}_\mathrm{c},\mathbf{r}_\mathrm{n}) & \text{if } d_{\mathrm{ol}}(\mathbf{r}_\mathrm{c},\mathbf{r}_\mathrm{n}) \geq d_{\mathrm{ol\_min}} \wedge d_{\mathrm{ol}}(\mathbf{r}_\mathrm{c},\mathbf{r}_\mathrm{n}) < d_{\mathrm{ol\_max}} \\ k_{\mathrm{pr}} d_{\mathrm{ol\_max}} \exp\left(\frac{d_{\mathrm{ol}}(\mathbf{r}_\mathrm{c},\mathbf{r}_\mathrm{n})}{d_{\mathrm{ol\_max}}} - 1\right) & \text{if } d_{\mathrm{ol}}(\mathbf{r}_\mathrm{c},\mathbf{r}_\mathrm{n}) \geq d_{\mathrm{ol\_max}} \\ 0 & \text{else} \end{cases} \tag{7}$$

We define the intercellular gap as the difference between the actual distance and the optimal distance.

$$d_{\mathrm{gap}}(\mathbf{r}_\mathrm{c},\mathbf{r}_\mathrm{n}) = \|\mathbf{v}_{\mathrm{nc}}\| - d_{\mathrm{opt}}(\mathbf{r}_\mathrm{c},\mathbf{r}_\mathrm{n}) \tag{8}$$

For computational convenience this gap is normalized as follows[9]:

$$\hat{d}_{\mathrm{gap}}(\mathbf{r}_\mathrm{c},\mathbf{r}_\mathrm{n}) = \begin{cases} \left|\sin\left(\frac{\pi}{2}\frac{d_{\mathrm{gap}}(\mathbf{r}_\mathrm{c},\mathbf{r}_\mathrm{n})}{(\delta_{\mathrm{adh}}-1)d_{\mathrm{seg\_cn}}}\right)\right| & \text{if } (1-\delta_{\mathrm{adh}})d_{\mathrm{seg\_cn}} < d_{\mathrm{gap}}(\mathbf{r}_\mathrm{c},\mathbf{r}_\mathrm{n}) < (\delta_{\mathrm{adh}}-1)d_{\mathrm{seg\_cn}} \\ 1 & \text{else} \end{cases} \tag{9}$$

We assume that stable cell-cell bonds form when the actual cell distance is in the range of $d_{\mathrm{adh}}(\mathbf{r}_\mathrm{c},\mathbf{r}_\mathrm{n})$ which is the product of the optimal distance and the adhesion distance coefficient $\delta_{\mathrm{adh}} = 1.3$. This corresponds to a stable cell-cell bond formation bridging a maximum cell gap of 5 to 6 µm[15].

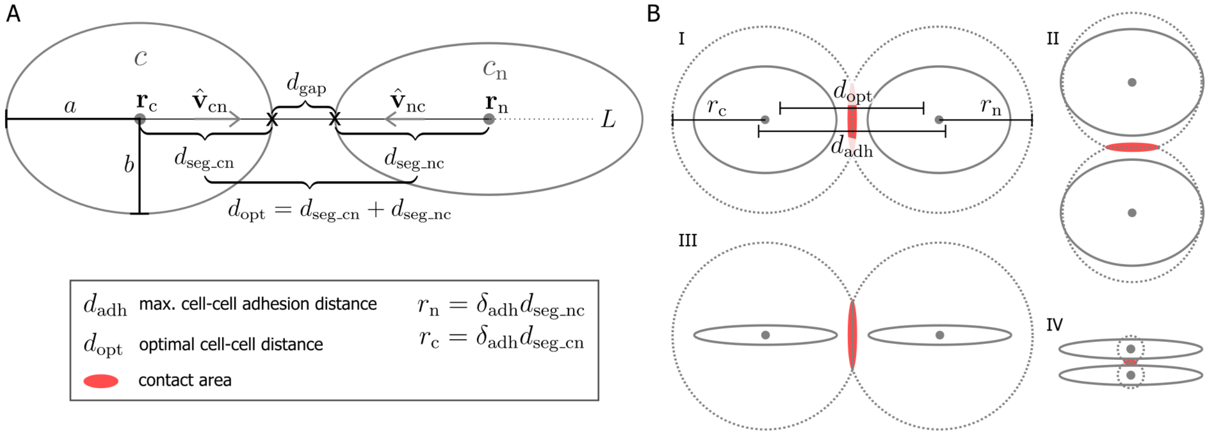

Supplementary Figure S2. Cell centre-based biomechanical model based on ellipsoids shown as 2D projection. (A) determination of the optimal cell distance d_{opt}. (B) Keratinocyte morphology is mimicked by ellipsoids. To determine the cell-cell adhesion force between two cells, their contact area has to be calculated. For computational convenience this is done with an approximation based on spheres[9] with radii r_{c} and r_{n}. While this yields quite accurate results for mildly irregular cells (I, II), this method over- or underestimates lateral adhesion to a large extent for flat cells (III, IV).

$$d_{\mathrm{adh}}(\mathbf{r}_{\mathrm{c}}, \mathbf{r}_{\mathrm{n}}) = \delta_{\mathrm{adh}} d_{\mathrm{opt}}(\mathbf{r}_{\mathrm{c}}, \mathbf{r}_{\mathrm{n}}) \tag{10}$$

The strength of cell-cell adhesion $F_{\mathrm{adh}}(\mathbf{r}_{\mathrm{c}}, \mathbf{r}_{\mathrm{n}})$ is correlated to the contact area of two adjacent cells. For computational convenience, we approximate the contact area by the contact area of the spheres with radii $r_{\mathrm{c}} = \delta_{\mathrm{adh}} d_{\mathrm{seg\_cn}}$ and $r_{\mathrm{n}} = \delta_{\mathrm{adh}} d_{\mathrm{seg\_nc}}$[9].

To determine the strength of cell-cell adhesion, we calculate the contact area of the adjacent cells. As the exact calculation of the contact area of adjacent ellipsoids would tremendously increase the computation time of the simulation, we approximate the contact area by the contact area of the spheres with radii r_{c} and r_{n}[9]:

$$A_{\mathrm{adh}}(\mathbf{r}_{\mathrm{c}}, \mathbf{r}_{\mathrm{n}}) = \frac{\pi}{4\left\|\mathbf{v}_{\mathrm{nc}}\right\|^2}\left(2\left\|\mathbf{v}_{\mathrm{nc}}\right\|^2 (r_{\mathrm{c}}^2 + r_{\mathrm{n}}^2) + 2r_{\mathrm{c}}^2 r_{\mathrm{n}}^2 - r_{\mathrm{c}}^4 - r_{\mathrm{n}}^4 - \left\|\mathbf{v}_{\mathrm{nc}}\right\|^4\right) \tag{11}$$

The radii are $r_{\mathrm{c}} = \delta_{\mathrm{adh}} d_{\mathrm{seg\_cn}}$ and $r_{\mathrm{n}} = \delta_{\mathrm{adh}} d_{\mathrm{seg\_nc}}$ (Supplementary Figure S2B). This approach of approximating the contact area yields accurate results for ellipses (ellipsoids) with a ratio of major to minor axis $\frac{a}{b}$ close to 1 (Supplementary Figure S2B, II). As cell shapes become more irregular the ratio becomes larger. For such irregularly shaped cells, the vertical contact area is underestimated whereas the horizontal contact area is overestimated (Supplementary Figure S2B III, IV). We take advantage of this approximation error to model the strong lateral cell-cell bonds of corneocytes, which have the most irregular cell shape[16].

The adhesion force $F_{\mathrm{adh}}(\mathbf{r}_{\mathrm{c}}, \mathbf{r}_{\mathrm{n}})$ of two neighbouring cells is the product of the contact area $A_{\mathrm{adh}}(\mathbf{r}_{\mathrm{c}}, \mathbf{r}_{\mathrm{n}})$, the normalized cell-cell gap, the linear spring constant $k_{\mathrm{adh}} = 2.2 \times 10^{-5}\mathrm{N\ m^{-1}}$[9] and the adhesion scaling factor k_{cn}.

$$F_{\mathrm{adh}}(\mathbf{r}_{\mathrm{c}}, \mathbf{r}_{\mathrm{n}}) = \begin{cases} k_{\mathrm{adh}} k_{\mathrm{cn}} (\hat{d}_{\mathrm{gap}}(\mathbf{r}_{\mathrm{c}}, \mathbf{r}_{\mathrm{n}}) A_{\mathrm{adh}}(\mathbf{r}_{\mathrm{c}}, \mathbf{r}_{\mathrm{n}})) & \text{if } d_{\mathrm{ol}}(\mathbf{r}_{\mathrm{c}}, \mathbf{r}_{\mathrm{n}}) < 0 \wedge \left\|\mathbf{v}_{\mathrm{nc}}\right\| < d_{\mathrm{adh}}(\mathbf{r}_{\mathrm{c}}, \mathbf{r}_{\mathrm{n}}) \\ 0 & \text{else} \end{cases} \tag{12}$$

Both forces, $F_{\mathrm{pr}}(\mathbf{r}_{\mathrm{c}}, \mathbf{r}_{\mathrm{n}})$ and $F_{\mathrm{adh}}(\mathbf{r}_{\mathrm{c}}, \mathbf{r}_{\mathrm{n}})$ are summed to the total cell-cell interaction force vector $\mathbf{F}(\mathbf{r}_{\mathrm{c}}, \mathbf{r}_{\mathrm{n}})$:

$$\mathbf{F}(\mathbf{r}_{\mathrm{c}}, \mathbf{r}_{\mathrm{n}}) = F_{\mathrm{pr}}(\mathbf{r}_{\mathrm{c}}, \mathbf{r}_{\mathrm{n}})\hat{\mathbf{v}}_{\mathrm{nc}} + F_{\mathrm{adh}}(\mathbf{r}_{\mathrm{c}}, \mathbf{r}_{\mathrm{n}})\hat{\mathbf{v}}_{\mathrm{cn}} \tag{13}$$

The adhesive force between basal cells and the basement membrane depends on the contact area between the particular cell and the membrane. This contact area is approximated with the contact area of a deformable sphere

with radius $r_{\rm c} = \delta_{\rm adh} d_{\rm opt}(\mathbf{r}_{\rm c}, \mathbf{r}_{\rm bm})$ and a plane with position vector $\mathbf{r}_{\rm bm}$ and normal vector $\hat{\mathbf{v}}_{\rm c\_bm}$ according to Kogut et al.[17]:

$$A_{\rm con}(\mathbf{r}_{\rm c}, \mathbf{r}_{\rm bm}) = \pi r_{\rm c}(r_{\rm c} - \|\mathbf{v}_{\rm c\_bm}\|) \tag{14}$$

$$F_{\rm adh}(\mathbf{r}_{\rm c}, \mathbf{r}_{\rm bm}) = \begin{cases} k_{\rm adh} k_{\rm c\_bm}(\hat{d}_{\rm gap}(\mathbf{r}_{\rm c}, \mathbf{r}_{\rm bm}) A_{\rm adh}(\mathbf{r}_{\rm c}, \mathbf{r}_{\rm bm})) & \text{if } \|\mathbf{v}_{\rm c\_bm}\| < d_{\rm adh}(\mathbf{r}_{\rm c}, \mathbf{r}_{\rm bm}) \\ 0 & \text{else} \end{cases} \tag{15}$$

with the same linear spring constant $k_{\rm adh}$ used in Eq. (12), the maximum adhesion distance $d_{\rm adh}(\mathbf{r}_{\rm c}, \mathbf{r}_{\rm bm}) = \delta_{\rm adh} d_{\rm opt}(\mathbf{r}_{\rm c}, \mathbf{r}_{\rm bm})$ (see also Eq. (10)), and $\hat{d}_{\rm gap}(\mathbf{r}_{\rm c}, \mathbf{r}_{\rm bm})$ according to Eq. (9). The gap between a basal cell and the basal membrane used in Eq. (9) is:

$$d_{\rm gap}(\mathbf{r}_{\rm c}, \mathbf{r}_{\rm bm}) = \|\mathbf{v}_{\rm c\_bm}\| - d_{\rm opt}(\mathbf{r}_{\rm c}, \mathbf{r}_{\rm bm}) \tag{16}$$

Finally, the total force $\mathbf{F}(\mathbf{r}_{\rm c})$ acting on cell c is:

$$\mathbf{F}(\mathbf{r}_{\rm c}) = F_{\rm adh}(\mathbf{r}_{\rm c}, \mathbf{r}_{\rm bm}) \hat{\mathbf{v}}_{\rm c\_bm} + \sum_{i=1}^{N} \mathbf{F}(\mathbf{r}_{\rm c}, \mathbf{r}_{{\rm n}_i}) \tag{17}$$

We numerically integrate the differential equation describing the cell movement of cell c over time [18]:

$$\mathbf{r}_{\rm c}(t + \Delta t) = \mathbf{r}_{\rm c}(t) + \frac{\Delta t}{\gamma} \mathbf{F}(\mathbf{r}_{\rm c}(t)) \tag{18}$$

The obtained results are numerically stable for $\Delta t = 36$ s (or smaller). The friction constant γ of epithelial cells in medium corresponds to 0.4 Ns m$^{-}1$[5].

Cell-cell-contact area calculation

The accuracy of the approach for approximating the cell-cell contact area $A_{\rm adh}$ depends on the ratio of the axes a/b as we assume $a = c$. The accuracy of the approximation is high for $a/b \approx 1$, but the accuracy is low for $a/b \gg 1$. This inaccurate approximation of the cell-cell contact area is convenient for scaling cell-cell-adhesion due to the intrinsic adhesion anisotropy of the epidermis. However, if the cell-cell contact area is used to scale or normalize intercellular exchange processes or contact-dependent processes like cell-cell contact mediated cell cycle control, this method cannot be applied due to the highly inaccurate results for irregular cell shapes. For such cells, contact area in vertical direction would be underestimated whereas the contact area in horizontal direction is overestimated.

Therefore, we set out to develop a computationally inexpensive new method to approximate the cell-cell contact area of ellipsoidal cells. In the following, the semi-principal axes of the cell ellipsoid and its adjacent neighbouring cell are $a_{\rm c}$, $b_{\rm c}$, $c_{\rm c}$ and $a_{\rm n}$, $b_{\rm n}$, $c_{\rm n}$, respectively. The two cells are located at $\mathbf{r}_{\rm c} = \begin{pmatrix} x_{\rm c} \\ y_{\rm c} \\ z_{\rm c} \end{pmatrix}$ and $\mathbf{r}_{\rm n} = \begin{pmatrix} x_{\rm n} \\ y_{\rm n} \\ z_{\rm n} \end{pmatrix}$. Moreover, we assume that the cells are in contact and the following condition holds: $\|\mathbf{v}_{\rm nc}\| \leq d_{\rm opt}(\mathbf{r}_{\rm c}, \mathbf{r}_{\rm n})$. The adjustable threshold $k_{\rm ratio\_max}$ is the ratio a/b above which a cell ellipsoid is considered to be highly irregular. We distinguish two cases for calculating the contact area $A_{\rm con}$ depending on the irregularity of two adjacent cells:

$$A_{\mathrm{con}}(\mathbf{r}_{\mathrm{c}}, \mathbf{r}_{\mathrm{n}}) = \begin{cases} A_{\mathrm{rec}}(\mathbf{r}_{\mathrm{c}}, \mathbf{r}_{\mathrm{n}}) \text{ if } a_{\mathrm{c}}/b_{\mathrm{c}} \geq k_{\mathrm{ratio\_max}} \vee a_{\mathrm{n}}/b_{\mathrm{n}} \geq k_{\mathrm{ratio\_max}} \\ A_{\mathrm{ell}}(\mathbf{r}_{\mathrm{c}}, \mathbf{r}_{\mathrm{n}}) \text{ else} \end{cases} \tag{19}$$

If one of both cells is highly irregular, the contact area A_{rect} is calculated based on the four following rectangles:

i. $R_{\mathrm{xy\_c}}$ with centre $(x_{\mathrm{c}}, y_{\mathrm{c}})$, width $2a_{\mathrm{c}}$ and height $2b_{\mathrm{c}}$
ii. $R_{\mathrm{xy\_n}}$ with centre $(x_{\mathrm{n}}, y_{\mathrm{n}})$, width $2a_{\mathrm{n}}$ and height $2b_{\mathrm{n}}$
iii. $R_{\mathrm{zy\_c}}$ with centre $(z_{\mathrm{c}}, y_{\mathrm{c}})$, width $2c_{\mathrm{c}}$ and height $2b_{\mathrm{c}}$
iv. $R_{\mathrm{zy\_n}}$ with centre $(z_{\mathrm{n}}, y_{\mathrm{n}})$, width $2c_{\mathrm{n}}$ and height $2b_{\mathrm{n}}$

Based on these four rectangles, we define the following two intersection rectangles:

i. $R_{\mathrm{int\_xy}}$ with width $w_{\mathrm{int\_xy}}$ and height $h_{\mathrm{int\_xy}}$ resulting from the intersection of rectangles $R_{\mathrm{xy\_c}}$ and $R_{\mathrm{xy\_n}}$
ii. $R_{\mathrm{int\_zy}}$ with width $w_{\mathrm{int\_zy}}$ and height $h_{\mathrm{int\_zy}}$ resulting from the intersection of rectangles $R_{\mathrm{zy\_c}}$ and $R_{\mathrm{zy\_n}}$

In the next step, we use the width and height of the intersection rectangles $R_{\mathrm{int\_xy}}$ and $R_{\mathrm{int\_zy}}$ to define the two radii

$$r_{\mathrm{xy}} = \begin{cases} w_{\mathrm{int\_xy}}/2 \text{ if } h_{\mathrm{int\_xy}} < b_{\mathrm{c}} \wedge h_{\mathrm{int\_xy}} < b_{\mathrm{n}} \\ h_{\mathrm{int\_xy}}/2 \text{ else} \end{cases} \tag{20}$$

and

$$r_{\mathrm{zy}} = w_{\mathrm{int\_zy}}/2 \tag{21}$$

Finally, the contact area between the two considered cells A_{rect} is the area of an ellipse with semi-axes r_{xy} and r_{zy}

$$A_{\mathrm{rec}}(\mathbf{r}_{\mathrm{c}}, \mathbf{r}_{\mathrm{n}}) = \pi r_{\mathrm{xy}} r_{\mathrm{zy}} \tag{22}$$

If the shape of both cells is not highly irregular according to the criteria defined above, the cell-cell contact area A_{con} is approximated in a way analogous to the method used to compute A_{adh} with a small modification. The radii of the two spheres ($\hat{r}_{\mathrm{c}}$ and $\hat{r}_{\mathrm{n}}$) used to approximate the contact area of the two cell ellipses are calculated in a different way. First, we determine the following two intersection ellipses:

i. intersection ellipse $E_{\mathrm{int\_c}}$ of cell ellipsoid E_{c} and plane P_{cn} with semi-axes $a_{\mathrm{int\_c}}$ and $b_{\mathrm{int\_c}}$
ii. intersection ellipse $E_{\mathrm{int\_n}}$ of cell ellipsoid E_{n} and plane P_{cn} with semi-axes $a_{\mathrm{int\_n}}$ and $b_{\mathrm{int\_n}}$

Plane P_{cn} is given by the point $\mathbf{r}_{\mathrm{c}}$ and the vectors $\mathbf{v}_{\mathrm{cn}}$ and $\begin{pmatrix}0\\1\\0\end{pmatrix}$ if $\mathbf{v}_{\mathrm{cn}} \neq \begin{pmatrix}0\\1\\0\end{pmatrix}$:

$$P_{\mathrm{cn}} = \{(x, y, z) \in \mathbb{R}^3 \mid x(z_{\mathrm{c}} - z_{\mathrm{n}}) + z(x_{\mathrm{n}} - x_{\mathrm{c}}) = x_{\mathrm{n}} z_{\mathrm{c}} - x_{\mathrm{c}} z_{\mathrm{n}}\} \tag{23}$$

Otherwise, P_{cn} is defined by $\mathbf{r}_{\mathrm{c}}$ and the vectors $\mathbf{v}_{\mathrm{cn}}$ and $\begin{pmatrix}1\\0\\0\end{pmatrix}$:

$$P_{\mathrm{cn}} = \{(x, y, z) \in \mathbb{R}^3 \mid y(z_{\mathrm{n}} - z_{\mathrm{c}}) + z(y_{\mathrm{c}} - y_{\mathrm{n}}) = y_{\mathrm{c}} z_{\mathrm{n}} - y_{\mathrm{n}} z_{\mathrm{c}}\} \tag{24}$$

Based on the intersection ellipses $E_{\mathrm{int\_c}}$ and $E_{\mathrm{int\_n}}$, the radii of the spheres used to approximate the cell-cell contact area are:

$$\hat{r}_{\mathrm{c}} = \frac{a_{\mathrm{int\_c}} b_{\mathrm{int\_c}}}{r_{\mathrm{c}}} \tag{25}$$

and

$$\hat{r}_{\mathrm{n}} = \frac{a_{\mathrm{int\_n}} b_{\mathrm{int\_n}}}{r_{\mathrm{n}}} \tag{26}$$

In this context $r_{\mathrm{c}} = \delta_{\mathrm{adh}} d_{\mathrm{seg\_cn}}$ and $r_{\mathrm{n}} = \delta_{\mathrm{adh}} d_{\mathrm{seg\_nc}}$ are analogous to the definition used for calculating A_{adh} in equation (11). In the next step, we calculate a scaled distance d_{cn} between the two spheres with radii $\hat{r}_{\mathrm{c}}$ and $\hat{r}_{\mathrm{n}}$.

Thus, we overcome the overestimation of lateral contact areas.

$$d_{\mathrm{cn}} = \frac{\|\mathbf{v}_{\mathrm{nc}}\| \left(\hat{r}_{\mathrm{c}} + \hat{r}_{\mathrm{n}}\right)}{r_{\mathrm{c}} + r_{\mathrm{n}}} \tag{27}$$

Finally, the contact area of the two ellipsoid cells is approximated as:

$$A_{\mathrm{ell}}(\mathbf{r}_{\mathrm{c}}, \mathbf{r}_{\mathrm{n}}) = \frac{\pi}{4d_{\mathrm{cn}}^2} \left(2d_{\mathrm{cn}}^2(\hat{r}_{\mathrm{c}}^2 + \hat{r}_{\mathrm{n}}^2) + 2\hat{r}_{\mathrm{c}}^2\hat{r}_{\mathrm{n}}^2 - \hat{r}_{\mathrm{c}}^4 - \hat{r}_{\mathrm{n}}^4 - d_{\mathrm{cn}}^4\right) \tag{28}$$

Semantic integration of BM and CBM

The physical properties of the microenvironment influence cellular decision-making; vice versa cells affect their surroundings *e.g.* by altering their shape or properties of the extracellular matrix[19]. EPISIM enables modelling such interactions by flexibly interweaving the BM and CBM wherever the model design requires[20]. In our epidermis model, we use this capability for several processes, including cell morphology change upon differentiation, cell-cell-adhesion, and corneocyte desquamation.

Biological exchange processes generally occur at membranes through specialized transporter proteins. To accurately describe the kinetics of an exchange reaction the surface area of the membrane must be taken into account. Thus, the BM calculates several parameters that can be accessed within a CBM: (i.) the contact area to the basement membrane, (ii.) the contact area between neighbouring cells, (iii.) the sum of the contact area to all neighbouring cells, (iv.) the surface area of a cell, and (v.) the total area that a single cell exposes at the surface of the tissue.

The adhesive properties of cells vary depending on their differentiation status[21]. To reflect this, we scale the strength of the cell-cell adhesion force accordingly.

Differentiation dependent cell-cell adhesion model

The mechanical strength of cell-cell bonds, *i.e.* the strength of cell-cell adhesion force F_{adh} is calculated according to equation (12). The adhesive force can be scaled by parameter k_{cn}. Pairwise biomechanical model parameters like k_{cn} are N-tuples with N being the number of adjacent neighbouring cells at a particular simulation step. Thus, k_{cn} can be mathematically expressed as $(k_{\mathrm{cn}_1}(t), \ldots, k_{\mathrm{cn}_N}(t))$ where $k_{\mathrm{cn}_i}(t)$ is the adhesion scaling parameter between cell c and its neighbouring cell n_i at simulation step t. This allows scaling the strength of the adhesive force F_{adh} between a particular cell and each individual neighbouring cell depending *e.g.* on the neighbouring cell's differentiation. The change of cell-cell adhesion controlled by $k_{\mathrm{cn}}(t)$ is modelled as follows:

$$k_{\mathrm{cn}}(t) = k_{\mathrm{cn}}(t-1) + k_1 - k_2 k_{\mathrm{cn}}(t-1) \tag{29}$$

In this model k_1 corresponds to the adhesion increase rate reflecting the production of new cell-adhesion proteins. The adhesion decrease rate k_2 is multiplied with the adhesion parameter of the previous simulation step

$k_{\mathrm{cn}}(t-1)$, to consider degradation of cell adhesion proteins due to homeostatic turnover. The increase and decrease terms of Eq. (29) have the steady state $\hat{k}_{\mathrm{cn}} = \frac{k_1}{k_2}$. Substituting:

$$k_{\mathrm{cn}}(t) = k_{\mathrm{cn}}(t-1) + k_2\hat{k}_{\mathrm{cn}} - k_2 k_{\mathrm{cn}}(t-1) \tag{30}$$

The adhesive properties of cells vary depending on their differentiation status, *e.g.* corneocytes contain corneodesmosomes[21]. Thus, the model incorporates distinct values of $\hat{k}_{\mathrm{cn}}$ for each possible pair of differentiation stages in the model (*e.g.* basal cell - spinous cell or basal cell - basal cell). Moreover, we use three different values for the adhesion decrease rate k_2 to model distinct scenarios: (i.) adhesion among viable keratinocytes (k_2), (ii.) adhesion among corneocytes during the maturation phase, and (iii.) adhesion among corneocytes during the desquamation phase ($k_{2\_\mathrm{c\_dec}}$).

The process of cornification entails a complex apoptotic pathway, which results in an increase of cell junctions, *e.g.* corneodesmosomes[21,22]. Afterwards, a cascade of enzymatic reactions involving proteases and their inhibitors orchestrate the gradual loss of adhesive junctions, and loose corneocytes desquamate at the surface[22]. In the model, we summarize these processes by subdividing the development of corneocyte adhesion in two phases, characterized by distinct values of the decrease rate k_2.

**model parameter**	**value**
δ_{ol}	0.15
$\delta_{\mathrm{ol\_max}}$	0.5
$d_{\mathrm{ol\_min}}$	0.1 µm
k_{pr}	2.2*10^{-3}N*m^{-1}
δ_{adh}	1.3
k_{adh}	2.2*10^{-5}N*m^{-1}
Δt	36 s
γ	0.4 Ns*m^{-1}
$\hat{k}_{\mathrm{cn}}$	0.01 (within SB, SB-SS), 0.03 (within SS), 0.02 (SS-SG),<br>0.1 (within SG), 0.08 (SG-SC), 0.15 (within SC)
$\hat{k}_{\mathrm{c\_bm}}$	0.01 (BM-SB)
k_2	0.04 (incr.) / 0.02 (decr.) for corneocytes<br>0.01 (viable keratinocytes)

Supplementary Table S2. Parameters of the cell-centre based biomechanical model (references given in the text)

Formal description of the epidermis *in silico* model

Models built with the EPISIM Platform are based on the modular multiscale model architecture underlying EPISIM. This model architecture allows decomposing multiscale models into reusable model entities. EPISIM semantically integrates these model entities during simulation using automatically generated connector components (MCCs)[20]. Any EPISIM model consists at least of a biomechanical model (BM) and a cell behavioural model (CBM). In a simulation step, the BM is simulated first, followed by the CBM (Supplementary Figure S3A).

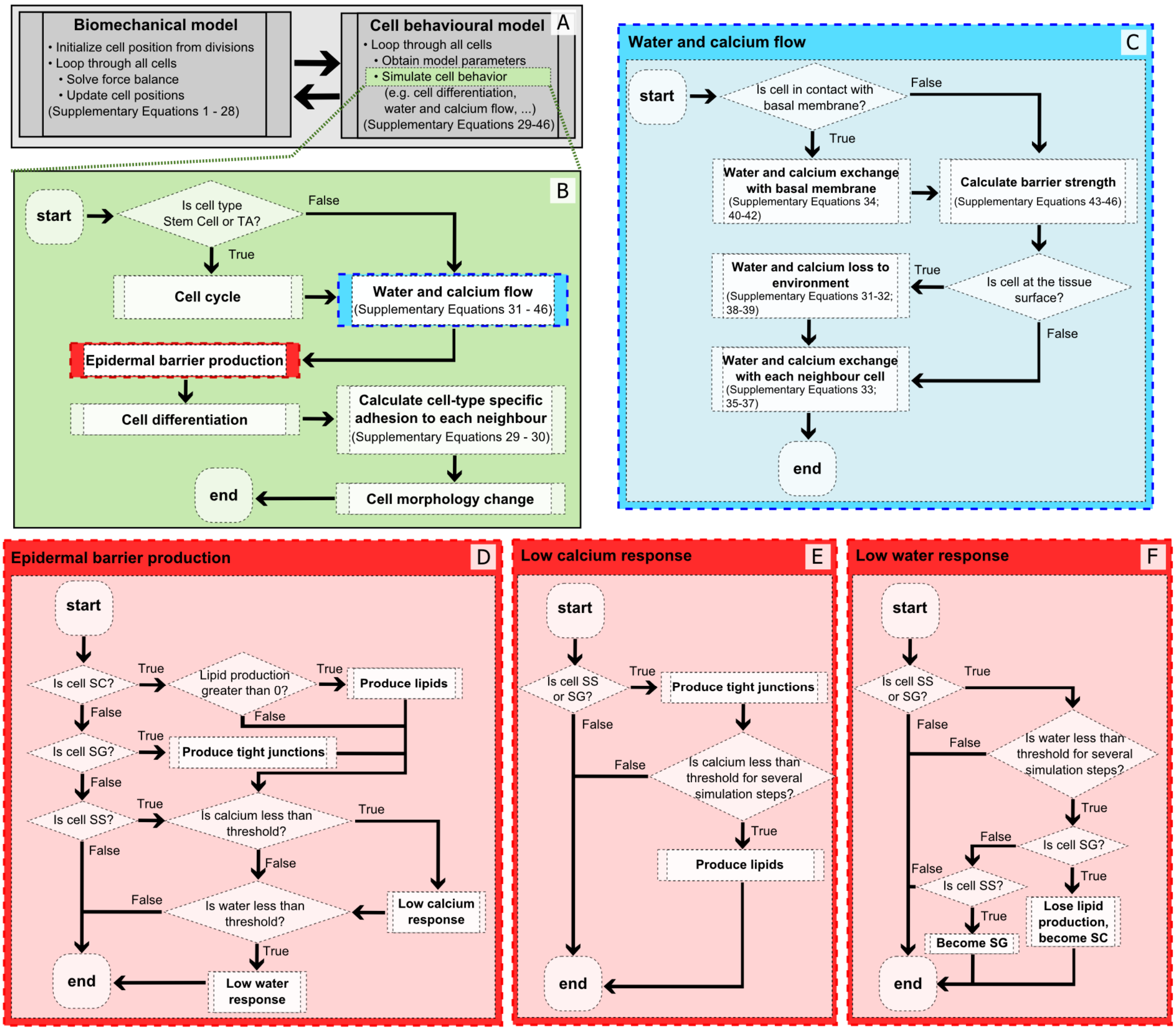

Supplementary Figure S3. Schematic overview of logical steps in the cell behavioural model. (A) In each simulation step, the biomechanical model is simulated first, followed by the cell behavioural model. (B) Summary of the processes simulated in each cell at every simulation step. In this work, we introduce submodels for water and calcium flow, and epidermal barrier production (highlighted in blue and red, respectively). Other submodels have been adapted from previous work[23,24]. (C) Diagram summarizing CBM for water and calcium flow. All the flow reactions are independent of cell type, and dependent on cell position in the tissue. (D) Diagram summarizing CBM for epidermal barrier production. Different cell types produce different barrier components. Low calcium or low water trigger more complex responses. (E) SS and SG cells react to transient low calcium levels by producing tight junctions. They react to sustained low calcium levels (i.e. over several simulation steps) by producing lipids. (F) Sustained low water levels lead to premature cell differentiation in SS and SG cells, a simplified representation of cell death due to water loss.

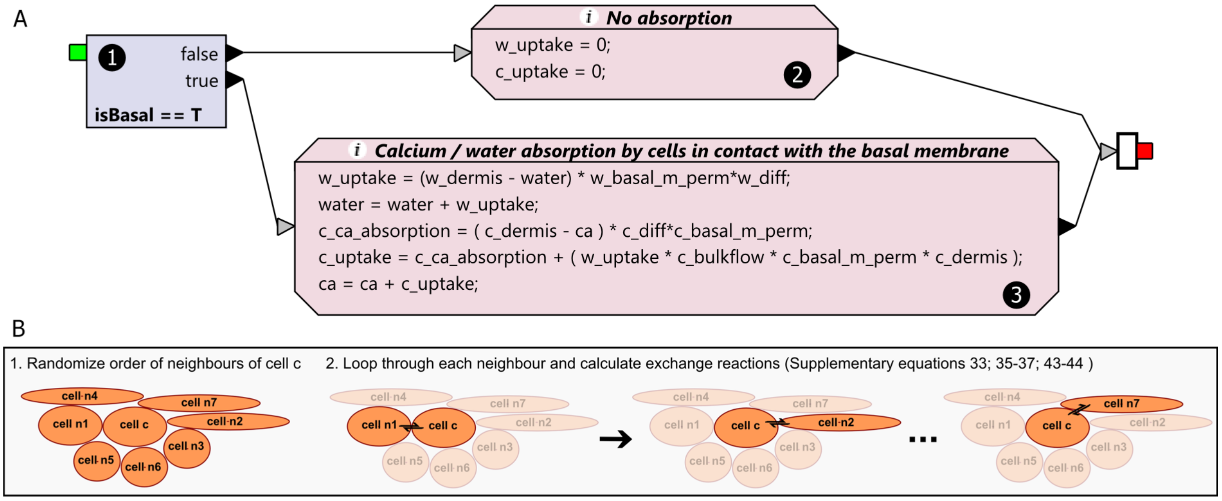

Supplementary Figure S4. EPISIM platform's graphical cell behavioural modelling language. (A) Realization of the water and calcium uptake of keratinocytes at the basal membrane: Only cells in contact with the basal membrane (1) take up water and calcium (3). The uptake of suprabasal cells is zero (2). Contact of a cell with the basal membrane is determined by the biomechanical model, and stored in the Boolean variable *isBasal*. (B) Schematic representation of algorithm for flow of molecules between cells. The cell currently being simulated (cell c) randomly selects each of its neighbours (cells n1 to n7) sequentially to calculate the barrier strength (equations(43)-(44)) and the water and calcium flow (equations (33), (35)-(37)).

We model cellular behaviour graphically with process diagrams using EPISIM Modeller's graphical modelling language[23]. Supplementary Figure S4 exemplarily shows how the water and calcium uptake by cells at the basal membrane is modelled with this approach. CBM process diagrams can be hierarchically structured into submodels. The processes modelled in the epidermis CBM are summarized in Supplementary Figure S3. In the following, we formally describe the mathematical equations underlying the graphical submodels of the epidermis CBM.

Flow of water and calcium

In the CBM submodel for flow of water and calcium (schematically depicted in Supplementary Figure S3C), we model exchange of molecular species at the level of the agents, *i.e.* flow occurs from cell to cell. As a side effect of this modelling choice, cell shape affects the speed of transport processes, *e.g.* a molecule will take longer to traverse a stack of many flat cells as opposed to a pile of a few rounded cells. This allows us to have a simplified representation of tissue tortuosity, which is important for SC barrier function[25]. We implement cell-to-cell transport by first looping through a cell's neighbours in random order, second calculating the magnitude of the flow to each individual neighbouring cell, and finally executing the transfer Supplementary Figure S4B. For example, suppose the amount of water of cell c at simulation step t is $\mathrm{W_c}(t)$; suppose further that this cell has only one neighbouring cell n_1 and that the diffusive flow of water results in a change in water content $\Delta\mathrm{W_c}(\mathrm{n_1}, t)$; then the water content of cell c at simulation step $t+1$ is $\mathrm{W_c}(t+1) = \mathrm{W_c}(t)\mathrm{W_c}(n_1, t)$. Cells in contact with the basal membrane – regardless of their cell type – calculate additional flow reactions to simulate transport from the dermis into the epidermis (and vice versa). Contact with the basal membrane is determined by the BM. Similarly, any cell at the tissue surface calculates additional flow reactions that simulate loss of water and calcium to the environment. Exposure to the tissue surface is determined by the BM: A cell is at the surface of the tissue if any of the voxels it encloses have the largest y-coordinate of all cells at a given position in the x-z plane. Each cell's water and calcium content is bound to the cell itself, not a particular position in the tissue. Thus, when a cell moves due to biomechanical forces in the BM computation, it takes all its contents with it (this includes water, calcium, lipids, and tight junctions).

We model three kinds of flow: water diffusion, calcium diffusion, and calcium transport. Water and calcium diffusion are calculated by adapting the diffusion equation to our discrete cell implementation (equations (33)-(34); (37); (41)). We implement calcium transport as flow coupled to the magnitude of water diffusion (equation (36)). Thus, calcium flow results from the sum of diffusive and water-coupled flow (equation (35)). The magnitude of all flow processes is damped by a term reflecting barrier strength; this is proportional to the lipid and tight junction content in a cell (for flow between a cell and the dermis/environment; equations (45)-(46)) or cell pair (for flow between cells; equations (43)-(44)).

Equations for water flow

$$\Delta \mathrm{W}_{\mathrm{evap}_{\mathrm{c}}}(t) = \mathrm{W}_{\mathrm{c}}(t) \cdot \mathrm{SR}_{\mathrm{c}}(t) \cdot B_{\mathrm{c}}(t, b_{\mathrm{TJ_W}}, b_{\mathrm{L_W}}) \cdot k_{\mathrm{evap}} \tag{31}$$

where $b_{\mathrm{TJ_W}}$ and $b_{\mathrm{L_W}}$ are parameters reflecting the contribution of tight junctions and lipids to the barrier against water flow, respectively. The cell's barrier strength $B_{\mathrm{c}}(t, b_{\mathrm{TJ_W}}, b_{\mathrm{L_W}})$ is restricted to the interval $[0, 1]$ (see also equation (45)). The surface exposure ratio $\mathrm{SR}_{\mathrm{c}}(t)$ is calculated by the BM and corresponds to the percentage of a cell's surface area that is at the tissue surface as determined by scanning for the largest y-coordinate in the x-z-plane. Thus, a cell that is only minimally exposed experiences only little water evaporation. We assume that k_{evap} is constituted by the sum of a constant basal term [26] with a term antiproportional to the ambient humidity H:

$$k_{\mathrm{evap}} = k_{\mathrm{H\_evap}} \cdot (1 - (\mathrm{H}/100)) + k_{\mathrm{b\_evap}} \tag{32}$$

The ambient humidity H is a global parameter that we allow to vary on an arbitrary scale from 0 to 100; importantly, it is always the same value for all cells. As a result of TEWL (equations (31)-(32)), cells at the surface of the tissue lose water to the environment. They replenish their water content through diffusion from neighbouring cells. The amount of water $\Delta \mathrm{W}_{\mathrm{c}}(\mathrm{n}_j, t)$ exchanged between a cell c and a neighbour cell n_j at simulation step t depends on their respective water content $\mathrm{W}_{\mathrm{c}}(t)$ and $\mathrm{W}_{\mathrm{n}_j}(t)$, the effective diffusion coefficient of water in the epidermis D_{W}, and the strength of the barrier to water between cell c and cell n_j, $B_{\mathrm{c}}(t, b_{\mathrm{TJ_W}}, b_{\mathrm{L_W}}, n_j)$

$$\Delta \mathrm{W}_{\mathrm{c}}(\mathrm{n}_j, t) = \left(\mathrm{W}_{\mathrm{c}}(t) - \mathrm{W}_{\mathrm{n}_j}(t)\right) \cdot D_{\mathrm{W}} \cdot B_{\mathrm{c}}(t, b_{\mathrm{TJ_W}}, b_{\mathrm{L_W}}, n_j) \tag{33}$$

This equation returns negative values if $\mathrm{W}_{\mathrm{c}}(t) < \mathrm{W}_{\mathrm{n}_j}(t)$, which we define as cell c receiving water from neighbour cell n_j. The barrier strength $B_{\mathrm{c}}(t, b_{\mathrm{TJ_W}}, b_{\mathrm{L_W}}, n_j)$ results from the average content of tight junctions and lipids of cells c and n_j, and is restricted to the interval $[0, 1]$ (see also equation (43)). Cells in contact with the basal membrane can obtain water through diffusion from the dermis, which we model as

$$\Delta \mathrm{W}_{\mathrm{c\_derm}}(t) = (\mathrm{W}_{\mathrm{derm}} - \mathrm{W}_{\mathrm{c}}(t)) \cdot D_{\mathrm{W}} \cdot k_{\mathrm{W\_perm}} \tag{34}$$

where $\mathrm{W}_{\mathrm{derm}}$ is a constant dermal water content, D_{W} the effective diffusion coefficient of water in the epidermis, and $k_{\mathrm{W\_perm}}$ a permeability constant for the basal membrane. Due to the assumptions that the environment constantly removes water and the basal membrane has a fixed concentration of water, we obtain a result analogous to a continuum source-sink model with the dermis acting as a source and the environment as a sink.

Equations for calcium flow

We assume that TEWL plays a major role in directing calcium transport, since changes in TEWL affect the epidermal calcium gradient[27–29]. Thus, we implement calcium transport as coupled to the magnitude of water flow; calcium can also diffuse. The total amount of calcium exchanged $\Delta \mathrm{Ca}_{\mathrm{c}}(\mathrm{n}_j, t)$ at simulation step t between

cell c and cell n_j results from the sum of water-coupled bulk flow and diffusive calcium flow multiplied by the barrier strength for calcium $B_{\mathrm{c}}(t, b_{\mathrm{TJ_{Ca}}}, b_{\mathrm{L_{Ca}}}, n_j)$:

$$\Delta\mathrm{Ca_c}(\mathrm{n}_j, t) = (\Delta\mathrm{Ca_{bulk}}(\mathrm{n}_j, t) + \Delta\mathrm{Ca_{diff}}(\mathrm{n}_j, t)) \cdot B_{\mathrm{c}}(t, b_{\mathrm{TJ_{Ca}}}, b_{\mathrm{L_{Ca}}}, n_j) \tag{35}$$

where $b_{\mathrm{TJ_{Ca}}}$ and $b_{\mathrm{L_{Ca}}}$ are parameters reflecting the contribution of tight junctions and lipids to the barrier against calcium flow, respectively. The barrier strength for calcium $B_{\mathrm{c}}(t, b_{\mathrm{TJ_{Ca}}}, b_{\mathrm{L_{Ca}}}, n_j)$ is restricted to the interval $[0, 1]$,and depends on the average amount of tight junctions and lipids of cells c and n_j (see also equation (44)). The amount of calcium exchanged by bulk flow $\Delta\mathrm{Ca_{bulk}}(\mathrm{n}_j, t)$ between cell c and cell n_j is coupled to the magnitude of water flow $\mathrm{W_c}(\mathrm{n}_j, t)$, scaled by a bulk flow constant k_{bulk}, and the calcium content $\mathrm{Ca_c}(t)$ of cell c

$$\Delta\mathrm{Ca_{bulk}}(\mathrm{n}_j, t) = \Delta\mathrm{W_c}(\mathrm{n}_j, t) \cdot \mathrm{Ca_c}(t) \cdot k_{\mathrm{bulk}} \tag{36}$$

The amount of calcium that moves by diffusion $\Delta\mathrm{Ca_{diff}}(\mathrm{n}_j, t)$ results from

$$\Delta\mathrm{Ca_{diff}}(\mathrm{n}_j, t) = \big(\mathrm{Ca_c}(t) - \mathrm{Ca_{n_j}}(t)\big) \cdot D_{\mathrm{Ca}} \tag{37}$$

where $\mathrm{Ca_c}(t)$ and $\mathrm{Ca_{n_j}}(t)$ are the calcium content of cell c and cell n_j, respectively, and D_{Ca} is the effective diffusion coefficient of calcium in the epidermis. We assume a cell c at the epidermal surface can lose calcium due to it becoming insoluble following water evaporation. The amount of calcium lost $\Delta\mathrm{Ca_{evap_c}}(t)$ at simulation step t is

$$\Delta\mathrm{Ca_{evap_c}}(t) = \Delta\mathrm{Ca_{c\_bulk}}(t) \cdot B_{\mathrm{c}}(t, b_{\mathrm{TJ_{Ca}}}, b_{\mathrm{L_{Ca}}}) \cdot k_{\mathrm{s\_loss}} \tag{38}$$

where $B_{\mathrm{c}}(t, b_{\mathrm{TJ_{Ca}}}, b_{\mathrm{L_{Ca}}})$ is the cell's barrier to calcium flow (see also (46)), and $k_{\mathrm{s\_loss}}$ is a proportionality constant. The amount of calcium lost due to bulk flow at the tissue surface $\Delta\mathrm{Ca_{c\_bulk}}(t)$ is

$$\Delta\mathrm{Ca_{c\_bulk}}(t) = \mathrm{W_{evap_c}}(t) \cdot \mathrm{Ca_c}(t) \cdot k_{\mathrm{bulk}} \tag{39}$$

Cells in contact with the basal membrane can also exchange calcium with the dermis both by diffusion and bulk flow. Thus, the amount of calcium $\Delta\mathrm{Ca_{c\_derm}}(t)$ that cell c obtains from the dermis is given by

$$\Delta\mathrm{Ca_{c\_derm}}(t) = (\Delta\mathrm{Ca_{derm\_bulk}}(t) + \Delta\mathrm{Ca_{derm\_diff}}(t)) \cdot k_{\mathrm{Ca\_perm}} \tag{40}$$

where $k_{\mathrm{Ca\_perm}}$ is the permeability of the basal membrane to calcium. The amount of calcium exchanged by diffusive flow $\Delta\mathrm{Ca_{derm\_diff}}(t)$ is given by

$$\Delta\mathrm{Ca_{derm\_diff}}(t) = (\Delta\mathrm{Ca_{derm}} - \mathrm{Ca_c}(t)) \cdot D_{\mathrm{Ca}} \tag{41}$$

where we assume that the calcium content of the dermis $\mathrm{Ca_{derm}}$ is constant. The amount of calcium exchanged by bulk flow $\Delta\mathrm{Ca_{derm\_bulk}}(t)$ is given by

$$\Delta\mathrm{Ca_{derm\_bulk}}(t) = \Delta\mathrm{W_{c\_derm}}(t) \cdot k_{\mathrm{bulk}} \cdot \mathrm{Ca_{derm}} \tag{42}$$

Epidermal barrier model

The corneocytes and the lipids in the stratum corneum are the main constituents of the epidermal barrier; the stratum granulosum also plays a role with tight junctions[30,31]. In the model, tight junctions are produced by SG cells, which are retained upon differentiation to SC cells. SC cells produce lipids. The processes modelled in the CBM of the epidermal barrier are summarized in Supplementary Figure S3(D-F). We assume that flow processes

occurring between a cell c and a neighbouring cell n_j in the epidermis (equations (33); (35)-(37)) and flow processes occurring between a cell c at the tissue surface and the environment (equations (31)-(32); (38)-(39)) are affected by the presence of lipids or tight junctions. More specifically, the content of lipids and tight junctions is used to calculate the barrier strength, which scales the magnitude of flow by a value restricted to the interval $[0,1]$. When a flow process occurs between a cell c and a neighbouring cell n_j, we take into account the average amount of lipids and tight junctions of c and n_j. When a flow process occurs between a cell c and the environment, we take into account only the lipids and tight junctions of c. The barrier strength also depends on model parameters $b_{\mathrm{TJ_{Ca}}}$, $b_{\mathrm{L_{Ca}}}$, $b_{\mathrm{TJ_W}}$, and $b_{\mathrm{L_W}}$, each having values restricted to the interval $[0,1]$. These model parameters reflect how lipids and tight junctions independently affect the flow of water and calcium to different extents, e.g. if $b_{\mathrm{TJ_{Ca}}} = 0.1$, then tight junctions can reduce the flow of calcium down to 10% of the unrestricted flow.

When flow occurs between a cell c and a neighbouring cell n_j, the barrier strength for water at simulation step t is given by

$$B_{\mathrm{c}}(t, b_{\mathrm{TJ_W}}, b_{\mathrm{L_W}}, n_j) = \left(1 - \left(\frac{\mathrm{TJ_c}(t) + \mathrm{TJ_{n_j}}(t)}{2\mathrm{TJ_{max}}}(1 - b_{\mathrm{TJ_W}})\right)\right) \cdot \left(1 - \left(\frac{\mathrm{L_c}(t) + \mathrm{L_{n_j}}(t)}{2\mathrm{L_{max}}}(1 - b_{\mathrm{L_W}})\right)\right) \quad (43)$$

where $b_{\mathrm{TJ_W}}$ and $b_{\mathrm{L_W}}$ are parameters reflecting the effect of tight junctions and lipids on water; $\mathrm{TJ_c}(t)$ and $\mathrm{TJ_{n_j}}(t)$ are the amount of tight junctions in cell c and cell n_j;$\mathrm{L_c}(t)$ and $\mathrm{L_{n_j}}(t)$ are the amount of lipids in cell c and cell n_j, respectively. The tight junction and lipid content are restricted to the interval $[0, \mathrm{TJ_{max}}]$, and $[0, \mathrm{L_{max}}]$, respectively, thus the terms $\frac{\mathrm{TJ_c}(t)+\mathrm{TJ_{n_j}}(t)}{2\mathrm{TJ_{max}}}$ and $\frac{\mathrm{L_c}(t)+\mathrm{L_{n_j}}(t)}{2\mathrm{L_{max}}}$ can only result in values in the interval$[0,1]$. Since we restrict the model parameters $b_{\mathrm{TJ_W}}$ and$b_{\mathrm{L_W}}$ to the interval $[0,1]$, the value of $B_{\mathrm{c}}(t, b_{\mathrm{TJ_W}}, b_{\mathrm{L_W}}, n_j)$ must also lie in the interval $[0,1]$. In particular, if both tight junctions and lipids are at their maximum attainable values in both cells, $B_{\mathrm{c}}(t, b_{\mathrm{TJ_W}}, b_{\mathrm{L_W}}, n_j)$ becomes the product of $b_{\mathrm{TJ_W}}$ and $b_{\mathrm{L_W}}$. The equation for the barrier strength for calcium is constructed analogously

$$B_{\mathrm{c}}(t, b_{\mathrm{TJ_{Ca}}}, b_{\mathrm{L_{Ca}}}, n_j) = \left(1 - \left(\frac{\mathrm{TJ_c}(t) + \mathrm{TJ_{n_j}}(t)}{2\mathrm{TJ_{max}}}(1 - b_{\mathrm{TJ_{Ca}}})\right)\right) \cdot \left(1 - \left(\frac{\mathrm{L_c}(t) + \mathrm{L_{n_j}}(t)}{2\mathrm{L_{max}}}(1 - b_{\mathrm{L_{Ca}}})\right)\right) \quad (44)$$

where $b_{\mathrm{TJ_{Ca}}}$ and $b_{\mathrm{L_{Ca}}}$ are parameters reflecting the effect of tight junctions and lipids on calcium, respectively. When flow occurs between a cell c and the environment, we take into account only the lipids and tight junctions of c. Thus, the equation for barrier strength for water becomes

$$B_{\mathrm{c}}(t, b_{\mathrm{TJ_W}}, b_{\mathrm{L_W}}) = \left(1 - \left(\frac{\mathrm{TJ_c}(t)}{\mathrm{TJ_{max}}}(1 - b_{\mathrm{TJ_W}})\right)\right) \cdot \left(1 - \left(\frac{\mathrm{L_c}(t)}{\mathrm{L_{max}}}(1 - b_{\mathrm{L_W}})\right)\right) \quad (45)$$

and the equation for barrier strength for calcium is

$$B_{\mathrm{c}}(t, b_{\mathrm{TJ_{Ca}}}, b_{\mathrm{L_{Ca}}}) = \left(1 - \left(\frac{\mathrm{TJ_c}(t)}{\mathrm{TJ_{max}}}(1 - b_{\mathrm{TJ_{Ca}}})\right)\right) \cdot \left(1 - \left(\frac{\mathrm{L_c}(t)}{\mathrm{L_{max}}}(1 - b_{\mathrm{L_{Ca}}})\right)\right) \quad (46)$$

**model parameter**	**value**
H	[0, 100]
D_{w}	0.75
$k_{\mathrm{H\_evap}}$	0.45
$k_{\mathrm{b\_evap}}$	0.29
$k_{\mathrm{W\_perm}}$	0.34
$\mathrm{W}_{\mathrm{derm}}$	70
k_{bulk}	0.12
$k_{\mathrm{s\_loss}}$	0.26
D_{Ca}	0.055
$k_{\mathrm{Ca\_perm}}$	0.3
$\mathrm{Ca}_{\mathrm{derm}}$	500
$b_{\mathrm{TJ}_{\mathrm{W}}}$	0.8
$b_{\mathrm{L}_{\mathrm{W}}}$	0.325
$b_{\mathrm{TJ}_{\mathrm{Ca}}}$	0.075
$b_{\mathrm{L}_{\mathrm{Ca}}}$	0.0098
$\mathrm{TJ}_{\mathrm{max}}$	200
$\mathrm{L}_{\mathrm{max}}$	200

Supplementary Table S3. Parameters of the cell behavioural model (Parameters chosen such that model simulations yield the described and discussed results)

Other Supplementary Figures S5-S10

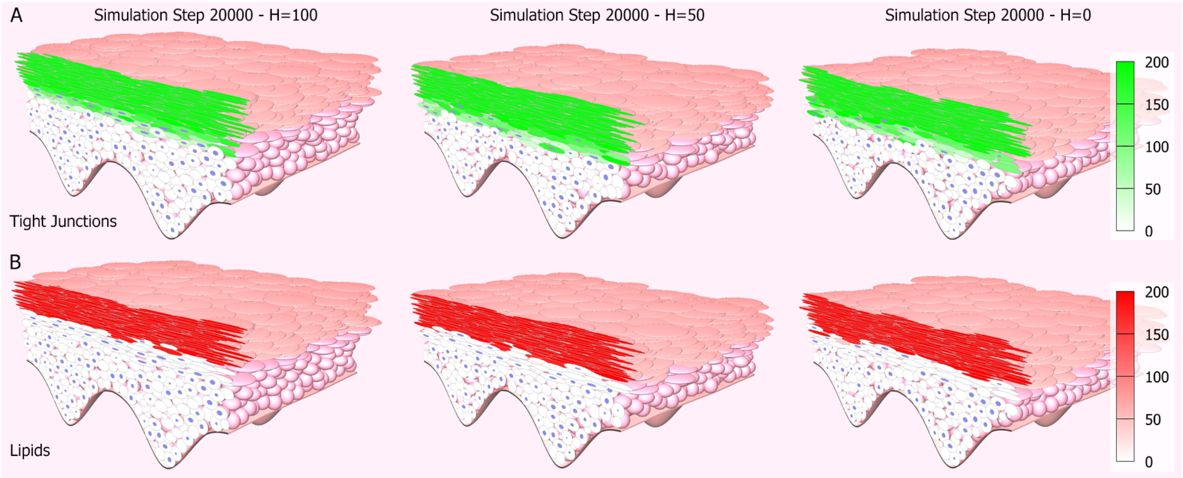

Supplementary Figure S5. Virtual cross-sections showing the barrier of homeostatic *in silico* epidermis. The epidermal barrier is modelled with the two components tight junctions and lipids. (A) Cells in the stratum granulosum express tight junctions, which individual cells retain after differentiation to stratum corneum cells. (B) Lipids are secreted in the stratum corneum. The transepidermal pattern of tight junction and lipid expression is the same for all H values.

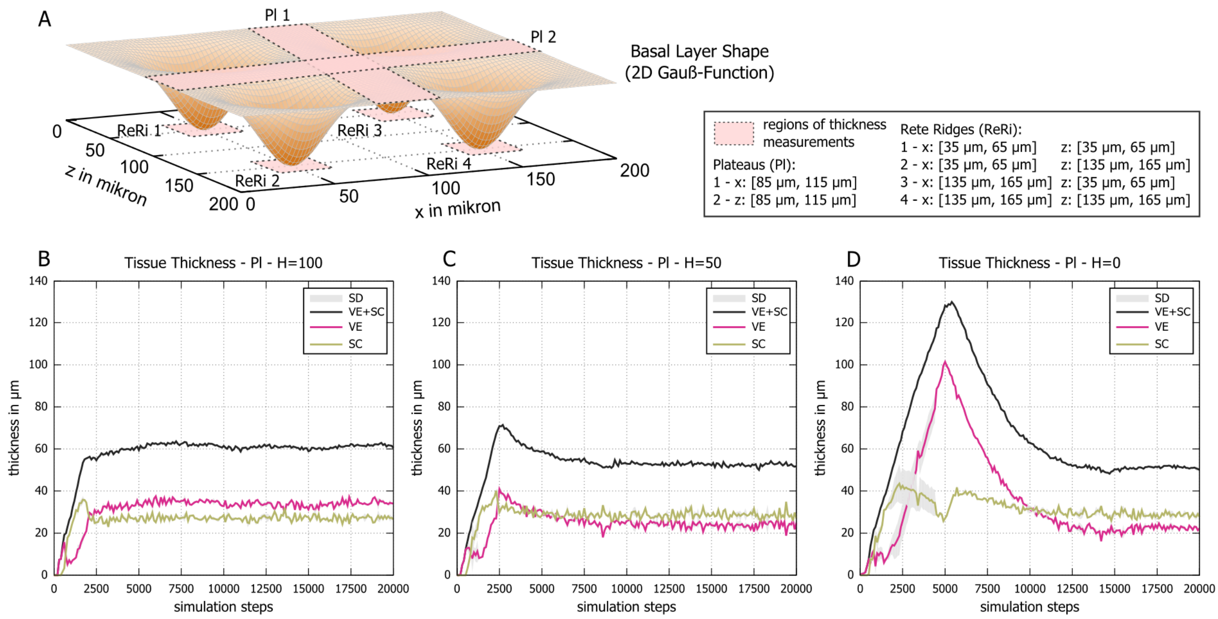

Supplementary Figure S6. Thickness measurements in different regions of the *in silico* epidermis. The undulated basal layer of the *in silico* epidermis is modelled with a 2D Gauß function. The total thickness of the whole *in silico* epidermis, the thickness of the viable epidermis (VE) as well as the thickness of the stratum corneum (SC) was measured over time in 6 subvolumes of the simulated tissue for one simulation run each. Four subvolumes are located in the rete ridges (ReRi). The two remaining subvolume thickness measurements reflect the thickness of the simulated tissue on the plateaus (Pl). Charts (B) - (D) show the averaged measurements taken in each of the three different simulation scenarios (high (H=100), medium (H=50) and low (H=0) ambient humidity) at Pl 1 and Pl 2 of the *in silico* epidermis. The averaged measurements of the four individual thicknesses taken in each rete ridge are shown in Figure 4 in the main article. The thickness of the *in silico* epidermis is nearly independent of the location (Pl 1 or 2) in all three simulation scenarios. However, the required number of simulation steps until homeostasis is reached varies significantly. A tremendously increasing initial overshoot in tissue thickness correlated to decreasing H values can be observed. *In vivo*, a transient tissue thickening is typical of the response to barrier injury[32,33]. Standard deviation (SD) in gray.

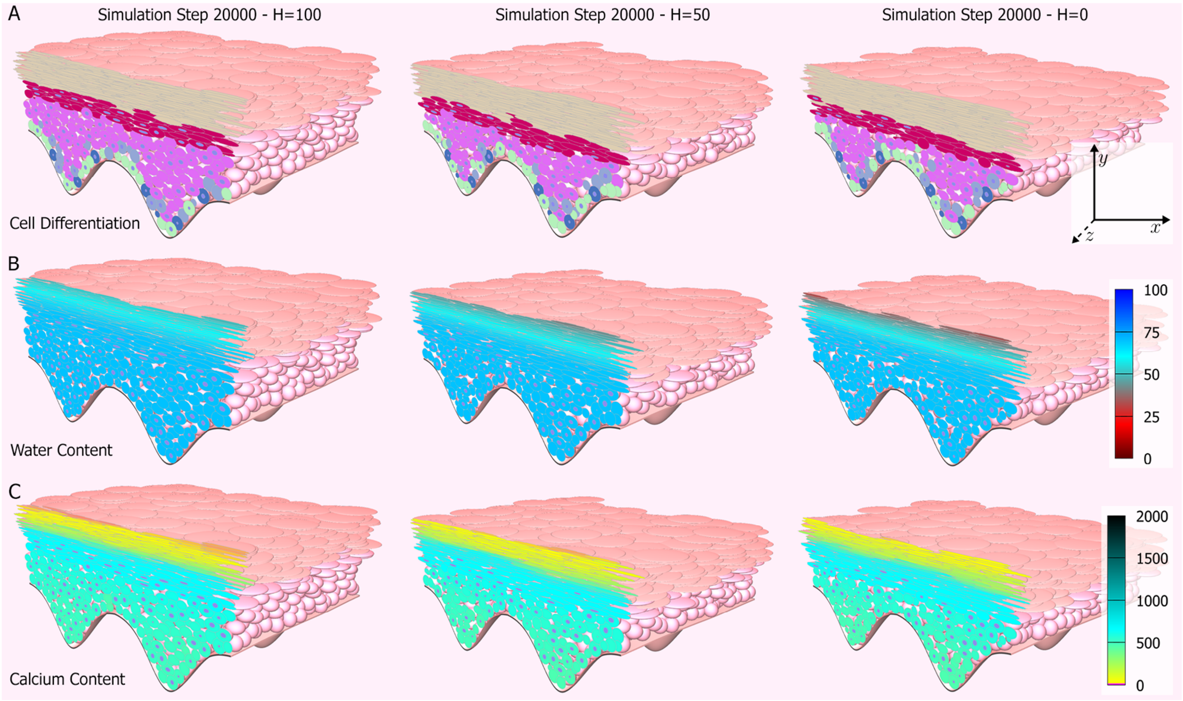

Supplementary Figure S7. Cross sections of homeostatic epidermal morphology for tissue simulations with high (H=100), medium (H=50) and low (H=0) ambient humidity. (A) Simulated keratinocytes stratify into the typical epidermal layers for all tested conditions. (B) During simulations, the transepidermal water gradient emerges dynamically from the described water flow and barrier model. Water content stays uniformly high in the viable layers, and only the corneocytes experience water loss in dry conditions. (C) Free calcium steadily increases within the viable epidermal layers, peaks in the stratum granulosum, and plummets in the stratum corneum, as corneocytes bind calcium ions.

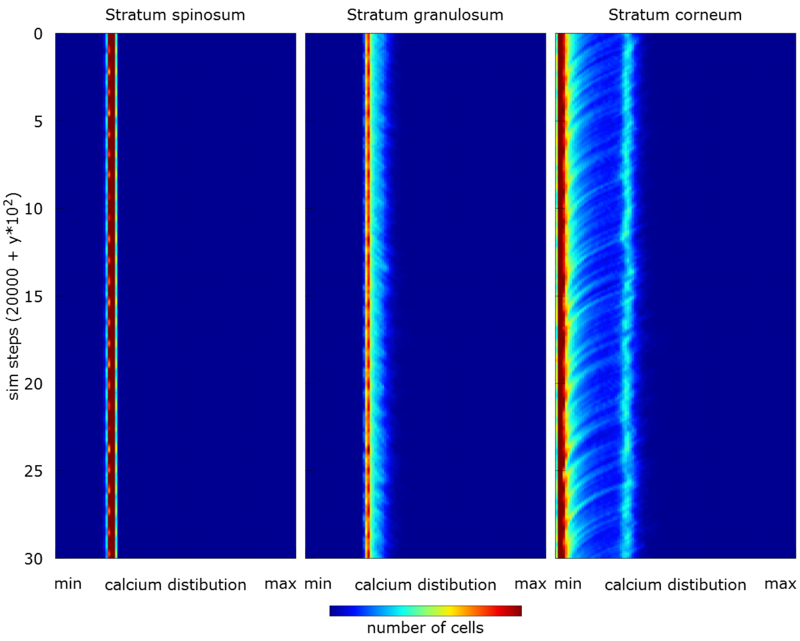

Supplementary Figure S8. Unchanged H value serving as control for the entire simulation time (3,000 simulation steps). We examined the effect of changing the set ambient humidity after a tissue simulation has reached homeostasis at maximum H. Initially, we simulated 20,000 simulation steps at H=100 to obtain a homeostatic *in silico* epidermis. We stored a tissue simulation snapshot at step 20,000 which was later on used to start three individual simulation runs. First, we continued to simulate with H=100 for additional 500 simulation steps to show that changes in the calcium distributions are not a result of relaunching the multi-agent-based simulation. The ambient humidity was then changed from maximum (H=100) to medium (H=50) and low (H=0) level at simulation step 20,500 (see Figure 5 in the main article).

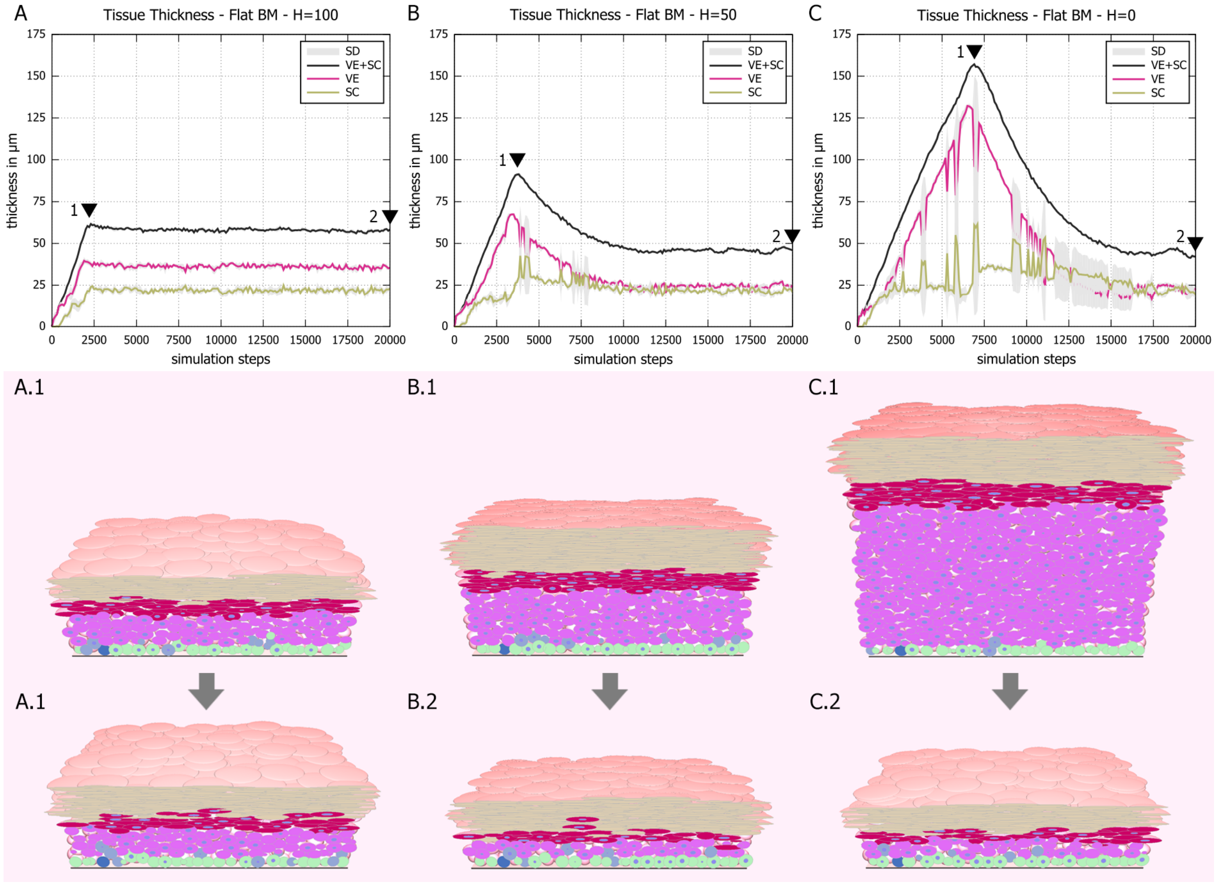

Supplementary Figure S9. Simulation with flat basement membrane qualitatively recapitulate model dynamics. (A) At H=100, the tissue immediately enters homeostasis. (A.1) Tissue cross-section at the timepoint indicated by black arrowhead 1, when the tissue has its maximum thickness. (A.2) Tissue cross-section at the timepoint indicated by black arrowhead 2, when the tissue has its homeostatic thickness. (B) At H=50, the tissue transiently overshoots its ideal thickness before becoming homeostatic. In contrast to simulation with an undulated basement membrane, there are stochastic variations in the tissue thickness measurements (spikes in standard deviation). These are caused by transiently disordered layers in some subvolumes. (B.1) Tissue cross-section at the timepoint indicated by black arrowhead 1, when the tissue has its maximum thickness. (B.2) Tissue cross-section at the timepoint indicated by black arrowhead 2, when the tissue has its homeostatic thickness. (C) At H=0, the tissue overshoots its ideal size dramatically, and converges to homeostasis with very slow dynamics. Frequency of transient layer disorder is increased (compare subpanel B, simulation at H = 50). The very thin tissue in homeostasis leads to increased probability of such stochastic events occurring. The homeostatic thickness is similar to simulations on undulated basement membrane when measured on the plateaus of the rete ridges (compare Supp. Fig. S5). *In vivo*, a transient tissue thickening is typical of the response to barrier injury[32,33]. (C.1) Tissue cross-section at the timepoint indicated by black arrowhead 1, when the tissue has its maximum thickness. (C.2) Tissue cross-section at the timepoint indicated by black arrowhead 2, when the tissue has its homeostatic thickness. All charts show averaged values from 6 subvolumes of the epidermis with standard deviation (SD) in gray.

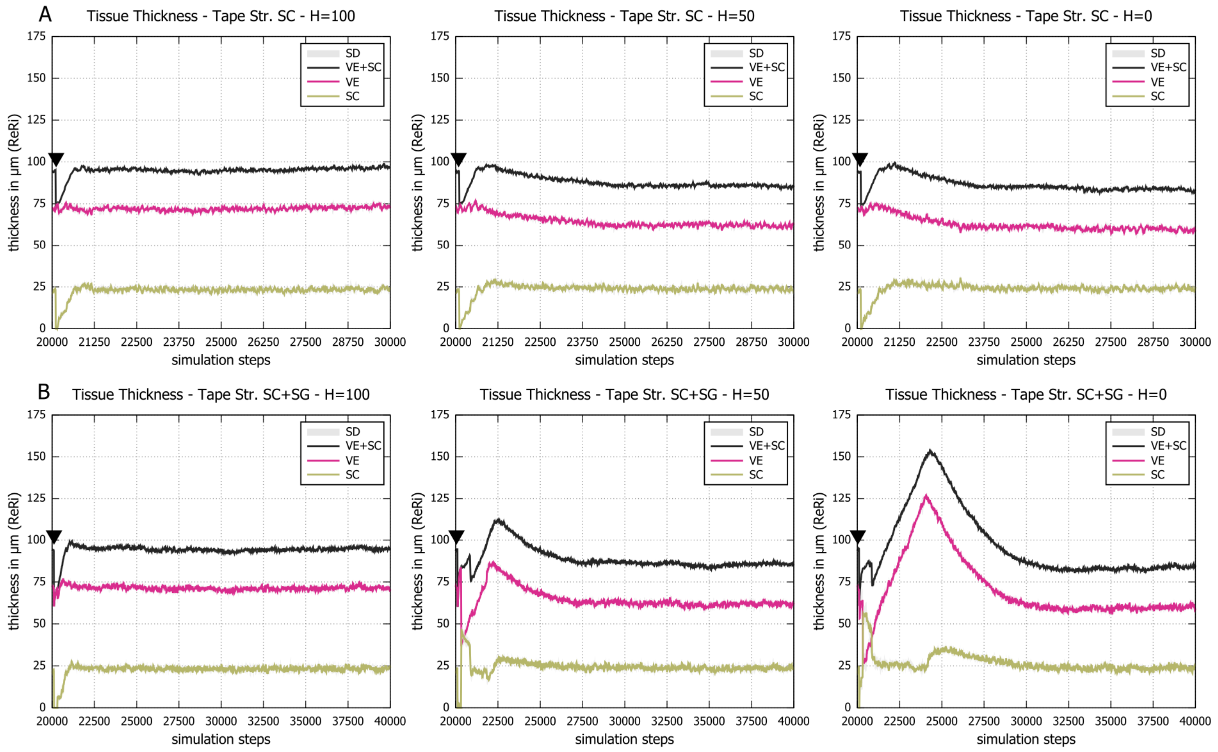

Supplementary Figure S10. *In silico* tape stripping experiments. (A) In each subpanel, a tissue simulation snapshot was loaded with the epidermis in homeostasis at simulation step 20000; simulation was continued for 100 simulation steps and then *in silico* tape stripping was performed by removing all SC cells in one simulation step (black arrowhead). The tissue very quickly re-enters homeostasis, regardless of ambient humidity after tape stripping. (B) In each subpanel, a tissue simulation snapshot was loaded with the epidermis in homeostasis at simulation step 20000; simulation was continued for 100 simulation steps and then *in silico* tape stripping was performed by removing all SG and SC cells in one simulation step (black arrowhead). The tissue exhibits transient thickening comparable to simulations growing from stem cells (compare Fig. 3 in the main text). All charts show averaged values from 4 subvolumes of the epidermis chosen at the Rete Ridges (compare Supplementary Figure S4) with standard deviation (SD) in gray.

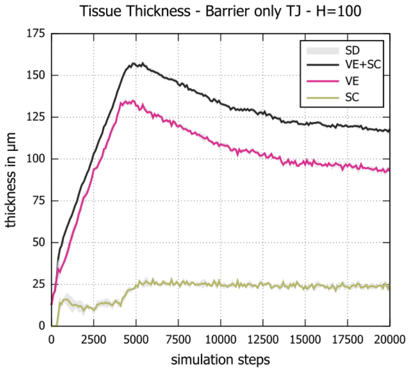

Supplementary Figure S11. Simulation with tight junctions (TJ) as the only barrier component. Averaged tissue thickness development over time for ambient humidity H=100 is shown for the rete ridges (see Supplementary Figure S6). The tissue lacking lipids as barrier component overshoots its final homeostatic thickness similar to model simulations with the full barrier at medium (H=50) and low (H=0) ambient humidity.

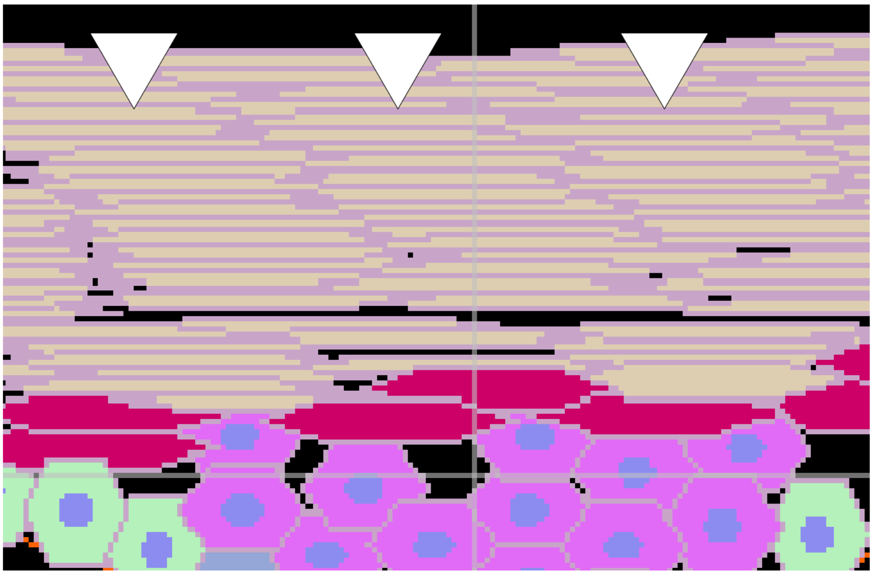

Supplementary Figure S12. Column-like stacking of corneocytes with adhesion proportional to contact area. During development of the biomechanical model, we performed test simulations with different corneocyte adhesion models. When using the contact area as scaling factor for adhesion strength, we observed stacking of corneocytes in columns (white arrowheads). However, human skin has an interdigitating corneocyte stacking pattern which simulations yielded for strong lateral adhesion (antiproportional to the contact area; see Figure 1A-II in the main manuscript).

Supplementary Video Legends

Supplementary Video S1

Supplementary Video S1 shows the development of the epidermal stratification during the first 5,000 simulation steps for humidity value H=100. Once homeostasis is reached the typical epidermal layering consisting of stratum basale (SB), stratum spinosum (SS), stratum granulosum (SG), and stratum corneum (SC) has evolved.

Supplementary Video S2

Supplementary Video S2 shows the development of the epidermal water gradient during the first 5,000 simulation steps for humidity value H=100. The transepidermal water gradient emerges dynamically during simulation. In homeostasis, the water content is constant in the viable layers and drops in the SC.

Supplementary Video S3

Supplementary Video S3 shows the development of the epidermal calcium gradient during the first 5,000 simulation steps for humidity value H=100. Free calcium constantly increases within the viable epidermal layers, peaks in the SG, and declines in the SC.

Supplementary Video S4

Supplementary Video S4 shows how changing the humidity from H=100 to H=0 during a simulation affects the tissue after it has achieved homeostasis at H=100 with a mature barrier. It can be observed that the water gradient in the SC gets steeper due to the increased evaporation at the epidermal surface.

References

1. Drasdo, D. in *Single-Cell-Based Model. Biol. Med.* (Anderson, A. A., Chaplain, M. J. & Rejniak, K.) 171–196 (Birkhäuser Basel, 2007). doi:10.1007/978-3-7643-8123-3_8
2. Dallon, J. in *Single-Cell-Based Model. Biol. Med.* (Anderson, A. A., Chaplain, M. J. & Rejniak, K.) 197–219 (Birkhäuser Basel, 2007). doi:10.1007/978-3-7643-8123-3_9
3. Walker, D. C. *et al.* The epitheliome: agent-based modelling of the social behaviour of cells. *Biosystems.* **76,** 89–100 (2004).
4. Adra, S., Sun, T., MacNeil, S., Holcombe, M. & Smallwood, R. Development of a three dimensional multiscale computational model of the human epidermis. *PLoS One* **5,** e8511 (2010).
5. Galle, J., Loeffler, M. & Drasdo, D. Modeling the effect of deregulated proliferation and apoptosis on the growth dynamics of epithelial cell populations in vitro. *Biophys. J.* **88,** 62–75 (2005).
6. Schaller, G. & Meyer-Hermann, M. A modelling approach towards epidermal homoeostasis control. *J. Theor. Biol.* **247,** 554–73 (2007).
7. Höhme, S. & Drasdo, D. A cell-based simulation software for multi-cellular systems. *Bioinformatics* **26,** 2641–2 (2010).
8. Li, X. *et al.* Skin Stem Cell Hypotheses and Long Term Clone Survival - Explored Using Agent-based Modelling. *Sci. Rep.* **3,** 1904 (2013).
9. Dallon, J. & Othmer, H. How cellular movement determines the collective force generated by the Dictyostelium discoideum slug. *J. Theor. Biol.* **231,** 203–22 (2004).
10. Palsson, E. & Othmer, H. A model for individual and collective cell movement in Dictyostelium discoideum. *Proc. Natl. Acad. Sci. U. S. A.* **97,** 10448–53 (2000).
11. Kashibuchi, N., Hirai, Y., O'Goshi, K. & Tagami, H. Three-dimensional analyses of individual corneocytes with atomic force microscope: morphological changes related to age, location and to the pathologic skin conditions. *Skin Res. Technol.* **8,** 203–11 (2002).
12. Koehler, M. J. *et al.* Keratinocyte morphology of human skin evaluated by in vivo multiphoton laser tomography. *Skin Res. Technol.* **17,** 479–86 (2011).
13. Palsson, E. A 3-D model used to explore how cell adhesion and stiffness affect cell sorting and movement in multicellular systems. *J. Theor. Biol.* **254,** 1–13 (2008).
14. Pathmanathan, P. *et al.* A computational study of discrete mechanical tissue models. *Phys. Biol.* **6,** 036001 (2009).
15. Walker, D. C., Georgopoulos, N. T. & Southgate, J. Anti-social cells: predicting the influence of E-cadherin loss on the growth of epithelial cell populations. *J. Theor. Biol.* **262,** 425–40 (2010).

16. Haftek, M. *et al.* Compartmentalization of the human stratum corneum by persistent tight junction-like structures. *Exp. Dermatol.* **20,** 617–21 (2011).

17. Kogut, L. & Etsion, I. Elastic-Plastic Contact Analysis of a Sphere and a Rigid Flat. *J. Appl. Mech.* **69,** 657 (2002).

18. Pitt-Francis, J. *et al.* Chaste: A test-driven approach to software development for biological modelling. *Comput. Phys. Commun.* **180,** 2452–2471 (2009).

19. DuFort, C. C., Paszek, M. J. & Weaver, V. M. Balancing forces: architectural control of mechanotransduction. *Nat. Rev. Mol. Cell Biol.* **12,** 308–19 (2011).

20. Sütterlin, T. & Grabe, N. in *Comput. Biophys. Ski.* (Querleux, B.) 421–460 (Pan Stanford Publishing, 2014). doi:10.4032/9789814463850

21. Candi, E., Schmidt, R. & Melino, G. The cornified envelope: a model of cell death in the skin. *Nat. Rev. Mol. Cell Biol.* **6,** 328–40 (2005).

22. Voegeli, R. & Rawlings, A. V. in *Treat. Dry Ski. Syndr.* (Lodén, M. & Maibach, H. I.) 149–178 (Springer Berlin Heidelberg, 2012). doi:10.1007/978-3-642-27606-4

23. Sütterlin, T., Huber, S., Dickhaus, H. & Grabe, N. Modeling multi-cellular behavior in epidermal tissue homeostasis via finite state machines in multi-agent systems. *Bioinformatics* **25,** 2057–63 (2009).

24. Sütterlin, T., Kolb, C., Dickhaus, H., Jäger, D. & Grabe, N. Bridging the scales: semantic integration of quantitative SBML in graphical multi-cellular models and simulations with EPISIM and COPASI. *Bioinformatics* **29,** 223–9 (2013).

25. Rawlings, A. V & Harding, C. R. Moisturization and skin barrier function. *Dermatol. Ther.* **17 Suppl 1,** 43–8 (2004).

26. Johnsen, G. K., Haugsnes, A. B., Martinsen, Ø. G. & Grimnes, S. Stratum corneum in vivo water content from TEWL-measurements. *Conf. Proc. ... Annu. Int. Conf. IEEE Eng. Med. Biol. Soc. IEEE Eng. Med. Biol. Soc. Annu. Conf.* **2008,** 3166–9 (2008).

27. Ahn, S. K., Hwang, S. M., Jiang, S. J., Choi, E. H. & Lee, S. H. The changes of epidermal calcium gradient and transitional cells after prolonged occlusion following tape stripping in the murine epidermis. *J. Invest. Dermatol.* **113,** 189–95 (1999).

28. Elias, P. M., Ahn, S. K., Brown, B. E., Crumrine, D. & Feingold, K. R. Origin of the epidermal calcium gradient: regulation by barrier status and role of active vs passive mechanisms. *J. Invest. Dermatol.* **119,** 1269–74 (2002).

29. Vicanová, J. *et al.* Normalization of epidermal calcium distribution profile in reconstructed human epidermis is related to improvement of terminal differentiation and stratum corneum barrier formation. *J. Invest. Dermatol.* **111,** 97–106 (1998).

30. Baroni, A. *et al.* Structure and function of the epidermis related to barrier properties. *Clin. Dermatol.* **30,** 257–62 (2012).

31. Kirschner, N. *et al.* Contribution of tight junction proteins to ion, macromolecule, and water barrier in keratinocytes. *J. Invest. Dermatol.* **133,** 1161–9 (2013).

32. Bargo, P. R., Walston, S. T., Chu, M., Seo, I. & Kollias, N. Non-invasive assessment of tryptophan fluorescence and confocal microscopy provide information on skin barrier repair dynamics beyond TEWL. *Exp. Dermatol.* **22,** 18–23 (2013).

33. Peppelman, M., van den Eijnde, W. A. J., Jaspers, E. J., Gerritsen, M.-J. P. & van Erp, P. E. J. Combining tape stripping and non-invasive reflectance confocal microscopy: an in vivo model to study skin damage. *Skin Res. Technol.* **21,** 474–84 (2015).
